# Value-modulated attentional capture depends on awareness

**DOI:** 10.3758/s13423-025-02734-1

**Published:** 2025-07-16

**Authors:** Francisco Garre-Frutos, Juan Lupiáñez, Miguel A. Vadillo

**Affiliations:** 1 Mind, Brain, and Behavior Research Center (CIMCYC), University of Granada, Granada, Spain; 2 Department of Experimental Psychology, University of Granada, Granada, Spain; 3 Department of Basic Psychology, Faculty of Psychology, Autonomous University of Madrid, Madrid, Spain

**Keywords:** Reward, Awareness, Learning, Attention, Capture

## Abstract

Value-modulated attentional capture (VMAC) refers to a process by which a priori neutral stimuli gain attentional priority when associated with reward, independently of goal or stimulus-driven attentional control. Although VMAC is considered an automatic and implicit process, the role of awareness of the stimulus-reward contingency on its learning process remains unclear at best. In a well-powered replication of a previous study, we found that VMAC is absent when participants are not explicitly informed about the stimulus-reward contingency in the pre-task instructions. In a second experiment, we show that when instructions are manipulated between groups, only the instructed group shows VMAC. Interestingly, although the no-instruction group did not show VMAC at the group level, participants who became aware of the stimulus-reward contingencies did nevertheless show robust VMAC at the end of the task. Meta-analytic evidence further supports our conclusion by showing that studies that include instructions about the stimulus-reward contingencies yield significantly larger VMAC effects. Taken collectively, these findings suggest that the learning process behind VMAC may not be entirely implicit.

**Supplementary Information:**

The online version contains supplementary material available at 10.3758/s13423-025-02734-1.

## Introduction

When a stimulus feature is predictive of reward, it biases attention regardless of physical properties or current goals (Awh et al., 2012; Theeuwes, 2018). In one of the first demonstrations of this phenomenon, Anderson et al. (2011a, b) adapted the additional singleton task (Theeuwes, 1992) to manipulate the value of two singleton distractors presented in a visual search task. Participants underwent a training stage where they had to search for two colors that predicted rewards of different magnitudes. In a test stage, the relevant feature changed, and participants were asked to search for a diamond-shaped stimulus instead, while the previous target colors acted as singleton distractors. Anderson et al. (2011a, b) found that when a high-value singleton distractor was presented in the search display, response times (RTs) increased compared to low-value singleton trials, an effect called “Value-Modulated Attentional Capture” (VMAC).^1^

In “two-stage” paradigms, as described above, the feature associated with reward receives higher attentional priority even though it is no longer task-relevant in the testing stage, which suggests a persistent and implicit attentional bias. Other paradigms have replicated the same findings even when color is always task-irrelevant and in designs where attending to the high-value distractor is detrimental to performance. Perhaps one of the best demonstrations has been provided by Le Pelley et al. (2015). In this study, they adapted the two-stage paradigm to make the reward-predictive feature task-irrelevant. Specifically, participants could earn larger rewards if they avoided looking at the singleton distractor, but critically, the color of the singleton distractor predicted the magnitude of the reward in each trial. With this setting, Le Pelley et al. (2015) showed that VMAC, despite color always being task-irrelevant and even when attending to the high-value distractor, contradicts instrumental goals.

Although there is ample evidence about the automaticity of VMAC once the effect is well consolidated, little is known about whether the learning process underlying the effect shares similar attributes. Prior research has shown that learning occurs when stimulus features are good predictors of high reward magnitude (Kim & Beck, 2020; Sali et al., 2014). However, whether awareness (explicit knowledge) of the stimulus-reward contingency is necessary for learning remains unclear. In the two-stage paradigm, participants are sometimes asked to guess the contingency between color and reward at the end of the test phase. Post hoc comparisons between “aware” and “unaware” participants often yield a null result (Anderson, 2013; 2015a, b; Anderson & Yantis, 2013; Grégoire & Anderson, 2019; Gregoire et al., 2021, 2022; Theeuwes & Belopolsky, 2012), leading to the conclusion that awareness has little influence on learning (Anderson, 2016; Anderson et al., 2021; Bourgeois et al., 2016; Failing & Theeuwes, 2018). Nevertheless, this conclusion might suffer from several issues. For instance, in the two-stage paradigm, the awareness test is often conducted at the end of the test phase. Unfortunately, this timing can lead to forgetting and interference when testing awareness of the memory traces learned during the training stages (Giménez-Fernández et al., 2020; Lovibond & Shanks, 2002), thus increasing the likelihood of erroneously classifying participants as “unaware.” In addition, it is known that inferences based on post hoc selection (i.e., selecting only unaware participants or comparing aware and unaware participants based on a post hoc awareness test) can be biased by measurement error (Shanks, 2017). Furthermore, although there is no evaluation of the reliability of awareness tests used in this specific literature, there is evidence that typical awareness tests tend to portray particularly low reliabilities (Vadillo et al., 2022).

Testing awareness immediately after the learning stage, as in the “one-stage” paradigm developed by Le Pelley et al. (2015), is less problematic. For instance, Experiment 2 of Le Pelley et al. (2015) measured awareness at the end of training, showing that most participants showed awareness of the color-reward contingencies. Critically, participants who reported being unaware of the contingencies also showed a significant VMAC effect, which led to the conclusion that learning VMAC does not need awareness. Again, one issue with the previous conclusion is the length of the training phase. In Experiment 2 of Le Pelley et al. (2015), participants performed three sessions on separate days, with 1,728 trials in total. It is known that when participants are instructed about the contingencies, VMAC can be observed after only 40 trials (Garre-Frutos et al., 2024), which makes it possible that those “unaware” participants became aware of the contingencies at the beginning of the training, but eventually forgot this in later stages of training, when awareness was tested. Additionally, in a similar study by Pearson et al. (2015), participants were not instructed about the feature-reward contingencies and were asked to report the contingency between color and reward at the end of the experiment. Although their results showed no significant association between contingency awareness and VMAC, at the group level, most of the participants showed high awareness,^2^ which makes it possible that most participants were too aware of the association at the end of the task to detect any meaningful association between awareness and VMAC.

The present study aimed to test the role of awareness about the color-reward contingency in VMAC in uninstructed participants. In both experiments, we used the same design and procedure employed in a previous study from our laboratory (Garre-Frutos et al., 2024), where VMAC was measured using much fewer learning trials compared to other studies that have measured awareness in the one-stage paradigm (Le Pelley et al., 2015; Pearson et al., 2015). If awareness of the feature-reward contingency plays a role in the learning of VMAC, we expect that the VMAC effect will be correlated with performance in the awareness test and also that pre-task instruction about these contingencies will modulate VMAC. To that aim, in Experiment 1, participants were not informed about the stimulus-reward contingencies, and we tested the association between VMAC scores and awareness at the end of training. In Experiment 2, we manipulated instructions given to participants in a between-group design to test the influence of such information before the learning stage. Finally, we conducted a meta-analysis on the literature to test the potential moderating effect of instructing participants about the contingency in the pre-task instructions.

## Experiment 1

Experiment 1 was a nearly exact replication of the learning stage of a previous study from our lab (Garre-Frutos et al., 2024), with the only exception that participants did not receive explicit instructions about stimulus-reward contingencies before conducting the task. At the end of the experiment, participants rated the points earned with each singleton color to assess their knowledge of the color-reward contingencies.

### Method

#### Participants

Based on a power analysis reported in the Online Supplementary Material, we aimed to recruit at least 80 participants. Potential participants were contacted through the distribution lists of the University of Granada. From the group of undergraduate students who showed an interest in participating, 83 participants (65 self-identified as female; *M*_age_ = 21.2 years; *SD*_age_ = 3.34 years) were recruited in exchange for course credits.^3^ All of them had normal or corrected-to-normal vision and were naïve as to the purpose of the experiment. Participants conducted the task in an online meeting with the investigator, where instructions regarding the calibration procedure were explained in detail (see below). We removed one participant for exceptionally low response accuracy (< 0.7).

#### Stimuli, design, and procedure

The materials and procedure of this experiment are based on Garre-Frutos et al. (2024). The study was conducted online, like a large body of research with this task (Albertella et al., 2019, 2020; Le Pelley et al., 2022; Liu et al., 2021; Watson et al., 2020). To control for differences in participants’ distance to the screen, we scaled the stimulus size to screen distance using the virtual chinrest developed by Li et al. (2020). Before starting the experiment, participants were asked to fit an object with a standard size (i.e., a credit card or a driver’s license) to a rectangle on the computer screen, the size of which they could change using two buttons from the keyboard. Then, participants performed a blind-spot procedure to estimate screen distance. They were asked to cover their right eye while looking with their left eye at a fixed placeholder that appeared in the center of the monitor while a red circle moved to the left. Participants were instructed to press the spacebar when they noticed that the circle disappeared. Screen distance was estimated by averaging five repetitions of this procedure.

**Fig. 1:**
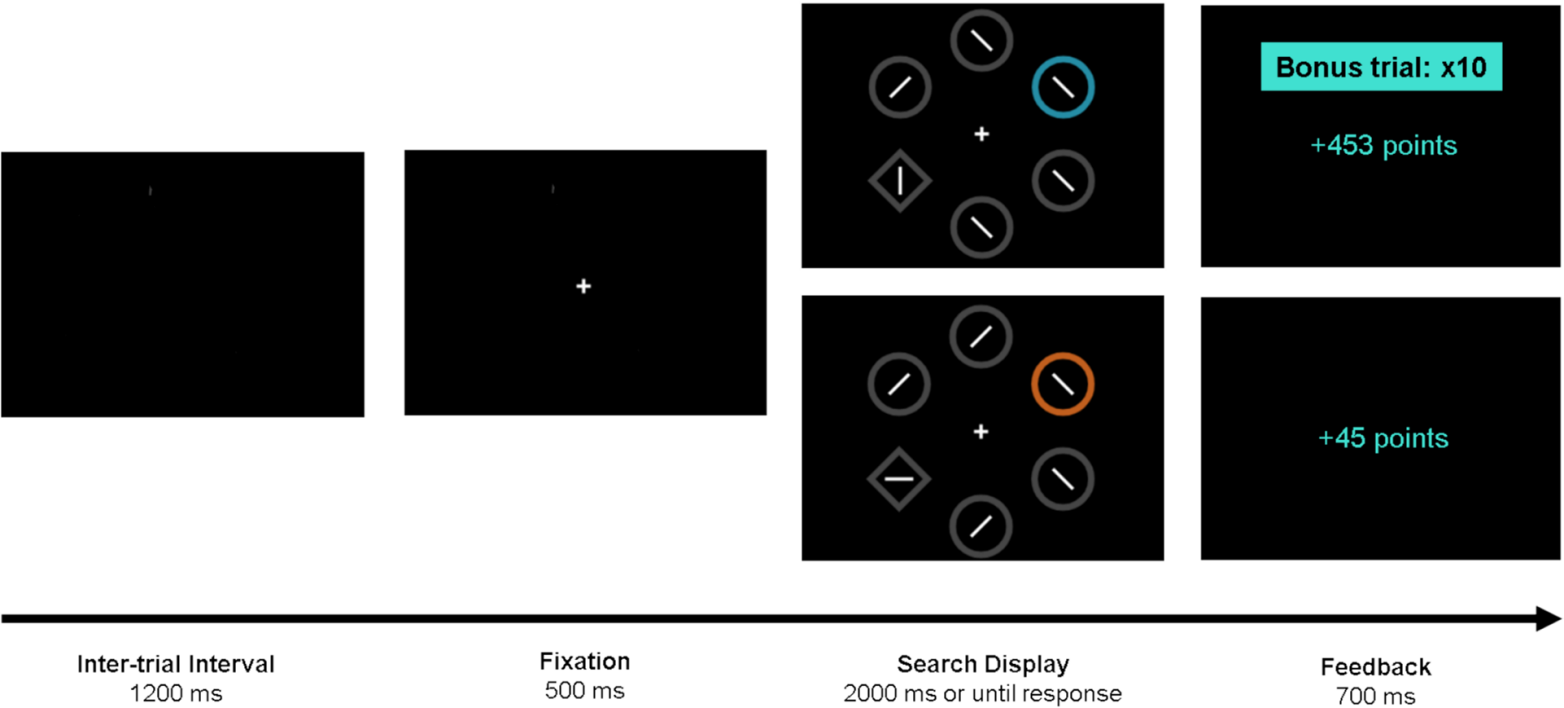
Example of the sequence of events in the experimental task. Participants could earn points based on performance, and when a high-value singleton appeared in the display, points were multiplied by 10. Feedback was provided in Spanish

The task was programmed in OpenSesame (Mathôt et al., 2012) and hosted in JATOS (Lange et al., 2015). A graphical representation of the procedure is presented in Fig. 1. Each trial started with a central fixation cross, followed by a search display containing six shapes (2.3° × 2.3° visual angle) evenly arranged around an imaginary circle (10.1º). Five shapes were circles, each containing a segment tilted 45° randomly to the left or right. The target was a diamond-shaped stimulus containing a segment oriented randomly horizontally or vertically. In most trials, one of the circles was colored, while the other shapes were gray. Participants could be assigned to three conditions of color pairs: blue and orange, green and pink, or red and yellow. The colors of the high- and low-reward distractors were randomly assigned. The location of the target and the distractor were random on each trial.

Participants were instructed to indicate, as quickly as possible, the orientation of the segment inside the diamond by pressing either ‘B’ for horizontal or ‘J’ for vertical, with faster responses earning more points. Each block included 24 trials, comprising ten trials with a distractor in the high-reward color (high-value condition), ten trials with a distractor in the low-reward color (low-value condition), and four distractor-absent trials (no distractor condition) where all shapes were gray. During the first part of the task (i.e., the rewarded phase), participants were awarded 0.1 points for every millisecond that their RTs were below 1,000 ms on low-value trials. On high-value trials, the points were multiplied by 10. Responses with RTs greater than 1,000 ms were awarded no points, and errors led to the loss of the same number of points that would have been earned. With this manipulation, participants distracted by the high-value singleton would acquire significantly fewer points, as the number of points earned in a given trial is directly proportional to performance. The search display remained on-screen until the participant responded or the trial timed out after 2,000 ms. Feedback was then provided for 700 ms, indicating the number of points won or lost for correct and incorrect responses. The inter-trial interval was 1,200 ms.

Immediately after the calibration described above, participants completed a brief practice phase consisting of 24 trials without singleton distractors. Following this, instructions informed participants that they could earn points based on their performance in the subsequent phase of the experiment. Participants were told that “faster and correct responses would yield more points, while incorrect responses would result in the loss of the same points that could be earned.” Importantly, they were not informed of the functional relationship between singleton color and reward. After reviewing the instructions, participants completed the task consisting of 12 blocks of trials.

Once participants completed the task, we asked them to provide a contingency rating to assess their knowledge of the stimulus-reward contingency. We presented participants with each color and the total number of points they had earned through the task. Then, participants were asked to write the absolute number of points that they believed they had earned when each singleton distractor had appeared on the screen. The order of presentation of the two singletons in the contingency rating test was counterbalanced. After the contingency rating test, participants conducted another task that was beyond the scope of the present work.

#### Data analysis

The analysis plan followed the same logic as in Garre-Frutos et al. (2024). We discarded incorrect responses (5.08%) and very fast or slow responses (RT < 150 ms or RTs > 1,800 ms; 0.08%), and we filtered RTs departing 2 *SD*s or more from each participant’s mean (4.25%).

We employed linear mixed-effects models to analyze how VMAC evolved over blocks. Specifically, we included as predictors Singleton (high-value, low-value, absent singleton), Block (1–12), and the Singleton × Block interaction. We set the contrast hypothesis matrix for the Singleton predictor to have two coefficients: one for the VMAC effect (high- vs. low-value) and the other for the attentional capture (AC) effect (low-value vs. absent singleton). We used repeated contrasts to set the hypothesis matrix, which allowed us to interpret the model intercept as the mean of overall RTs. Following Barr et al. (2013), we fitted the maximal random effect structure derived from our research design supported by our data (Bates et al., 2018; Matuschek et al., 2017). As in Garre-Frutos et al. (2024), data were best fitted by a power function, so we decided to log-scale the Block predictor and RTs to approximate a power function. Following the notation of the lme4 R package (Bates et al., 2015), the model’s formula for our maximal model is:

$$log\left(RT\right)\sim Singleton\hspace{0.33em}\times \hspace{0.33em}log(Block)\hspace{0.33em}+(Singleton\hspace{0.33em}\times \hspace{0.33em}log(Block) |\hspace{0.33em}Participant)$$

The analysis of response accuracy followed the same rationale as that for RTs, except that we employed a mixed logistic regression model instead (Jaeger, 2008), and the Block predictor was not log-scaled. For all models, we also report ANOVA-like tables in the Online Supplementary Materials.

We employed non-parametric tests for mean comparisons or correlations whenever non-normality was detected to analyze the contingency rating. Given that correlational research is highly influenced by measurement error, in the Online Supplementary Material, we also report a reliability multiverse analysis^4^ of the VMAC effect (high vs. low value) over a plausible range of data preprocessing specifications (as in Garre-Frutos et al., 2024). To assess the potential impact of reliability, we also corrected the observed correlation coefficient by the reliability of VMAC using Spearman’s disattenuation formula (*r*_corrected_). As we cannot know to what extent the awareness test is contaminated by measurement error (Franco-Martínez et al., 2025), we assumed that its reliability is at least “acceptable” (*r*_*xx’*_ = 0.7; Nunnally, 1978), even though there is evidence suggesting that commonly employed awareness tests portray much lower reliabilities (see Vadillo et al., 2020b).

Lastly, we computed Bayes factors (*BF*s) using the BayesFactor R package (Morey & Rouder, 2024) to gather evidence in favor of the null or alternative hypothesis for the VMAC contrasts using the default priors typically employed in commercial software. We interpreted *BF*s using the conventional thresholds provided by Van Doorn et al. (2021). We use subscripts to indicate whether the value of the BF supports the null hypothesis (*BF*_*01*_) or the alternative hypothesis (*BF*_*10*_).

### Results

For RTs (see Table 1), only the low-absent contrast and the Block predictor were significant, showing an AC effect (*M*_AC_ = 16.60, 95% confidence interval (CI) [11.4, 21.83]) and a decrease in RTs across the task. Interestingly, the VMAC effect was non-significant (*M*_VMAC_ = 1.39, 95% CI [−4.15, 6.93], *BF*_*01*_ = 7.61). Regarding accuracy, the only significant predictor was Block, reflecting that accuracy increased across blocks.

**Table 1:** Model summaries for the selected models for response times (RTs) and accuracy in Experiment 1

	RTs			Accuracy		
*Predictors*	*Estimates*	*CI*	*p*	*Odds ratios*	*CI*	*p*
(Intercept)	6.462	6.435–6.488	**<0.001**	25.341	21.223–30.257	**<0.001**
VMAC	0.002	−0.006–0.011	0.589	1.042	0.916–1.186	0.633
AC	0.025	0.017–0.033	**<0.001**	0.911	0.6765–1.085	0.297
Block	−0.036	−0.042 to −0.029	**<0.001**	1.126	1.030–1.230	**0.009**
VMAC × Block	0.003	−0.002–0.008	0.280	1.008	0.886–1.146	0.906
AC × Block	−0.006	−0.012–0.001	0.097	1.008	0.848–1.200	0.924
**Random effects**						
σ^2^	0.031			3.290		
τ_00_	0.015 _Intercept_			0.535 _Intercept_		
τ_11_	0.001 _VMAC_					
	0.0003 _AC_					
	0.001 _Block_			0.035 _Block_		
ρ_01_	0.022			0.052 _Intercept_		
	0.349					
	−0.274					
ICC	0.343			0.148		
N	82			82		
Observations	21,445			23,554		
Marginal R^2^/Conditional R^2^	0.030/0.362			0.004/0.151		

*Note*. Bold entries denote statistical significance. *p*-values were computed using Satterwhite correction

CI = confidence interval, ICC = intraclass correlation coefficient, τ = random effects, ρ = correlation between random effects

**Fig. 2:**
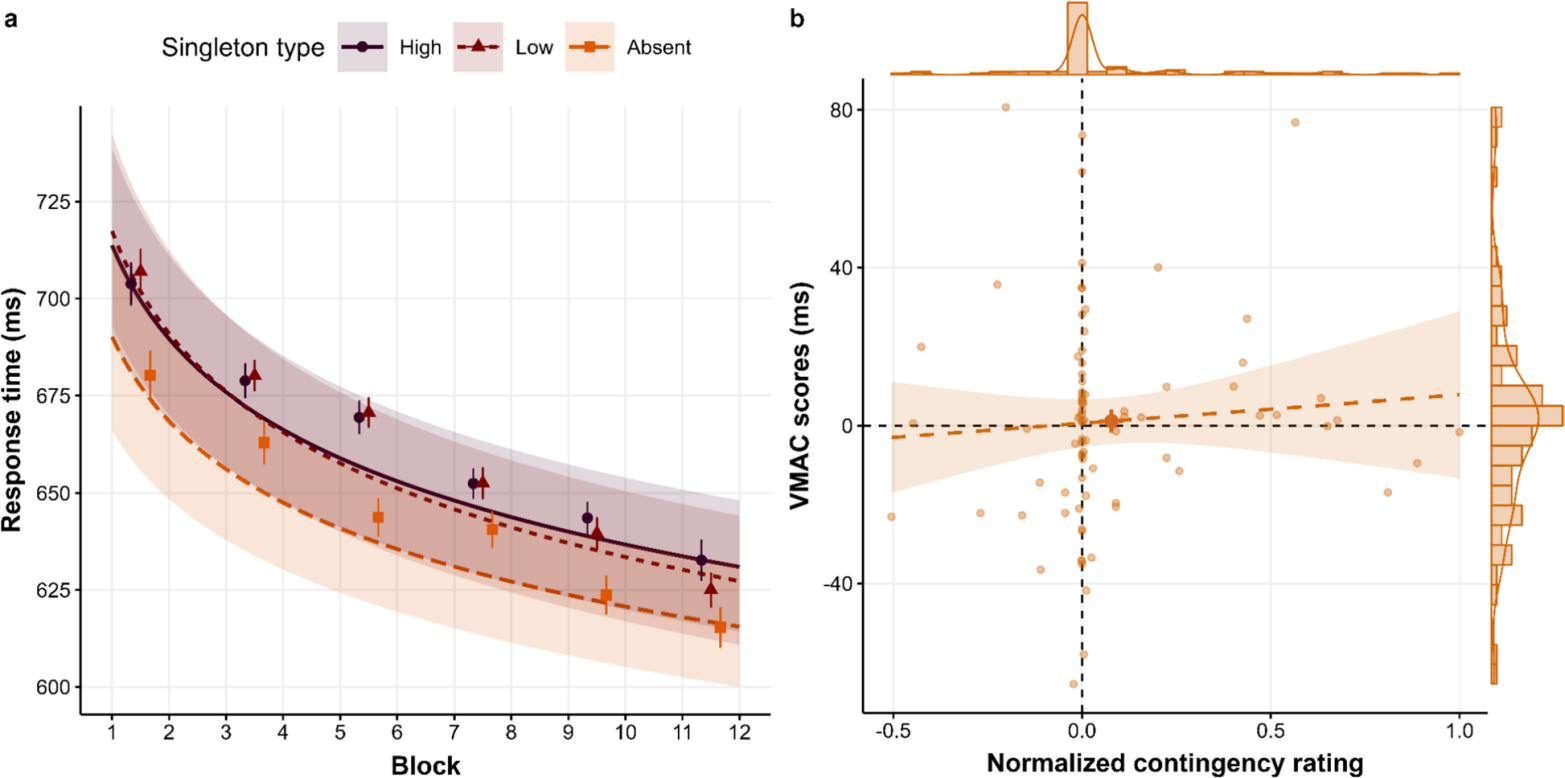
(**a**) Model predictions as a function of singletons across blocks. Lines represent the predicted conditional mean in the response scale, while shaded areas indicate the 95% confidence interval (CI). Raw mean response times (RTs) using epochs of two blocks are indicated by dots, and error bars represent the standard error of the mean (SEM). (**b**) Correlation between the value-modulated attentional capture (VMAC) effect and the normalized contingency rating. The line intervals represent the linear prediction of the VMAC effect as a function of the contingency rating, while the shaded area is the CI. The margin shows the marginal distribution (histogram and density), and the darker filled dot represents the mean of VMAC and the contingency rating at the group level, while error bars indicate SEM

For contingency ratings, the estimated difference in points between the high- and low-value singletons was significantly different from zero (*V* = 1,270.5, *p* = 0.019; *M*_high-low_* =* 3,478.9*, SD*_high-low_* =* 11,861.12). Nevertheless, only 40 participants (48.78%) estimated that the high-value singleton led to more points than the low-value singleton, and the median estimated difference was 0. In other words, the significant difference seems to be driven by a few participants guessing the correct contingency. No significant correlation was observed between the VMAC effect and contingency ratings (*r* = 0.12, *p* = 0.29, *r*_Corrected_ = 0.17; Fig. 2b).

### Discussion

Experiment 1 replicated the classical attentional capture effects typically observed in the additional singleton task (Theeuwes, 1992, 1994). However, despite the strong attentional capture effect, we did not observe a VMAC effect, even though participants were extensively exposed to the critical feature-reward contingencies. Moreover, contingency estimation did not significantly correlate with the VMAC effect. Our inability to find a significant association could represent a real null association between VMAC and awareness, but it could also be due to the specific awareness measure employed in this study. Although the reliability of VMAC was acceptable by psychometric standards, we cannot know to what extent our awareness measure is contaminated by measurement error. The absence of VMAC in Experiment 1 is particularly revealing if we consider that the main difference with Garre-Frutos et al. (2024) is the omission of explicit instructions about the feature-reward contingency. This result is consistent with other studies, where removing instructions about stimulus-reward contingency abolished VMAC and only “aware” participants showed the effect (Failing & Theeuwes, 2017; Le Pelley et al., 2017; Meyer et al., 2020; see also Le Pelley et al., 2019), which suggests that instructed learning may affect the learning process underlying VMAC.

## Experiment 2

Previous studies have explored the role of awareness or instructions, comparing the results of different experiments or the VMAC scores of “aware” and “unaware” participants based on post hoc selection using awareness tests. To our knowledge, only Pearson et al. (2015) manipulated the content of the instructions that participants receive before the learning stage in the one-stage paradigm. Nevertheless, Pearson et al. (2015) manipulated whether participants knew in advance that directing gaze toward the distractor would result in the omission of reward. Therefore, in Experiment 2, we manipulated the content of instructions regarding the stimulus-reward contingencies in a between-group design. In one group, participants were informed in advance about the role of the singleton color in the number of points they could earn, as in Garre-Frutos et al. (2024), while another group of participants performed the task without this information about the stimulus-reward contingency, as in Experiment 1.

### Method

#### Participants

We recruited 165 participants (148 self-identified as female; *M*_age_ = 20.3 years; *SD*_age_ = 6.05 years) from the University of Granada and the Autonomous University of Madrid. Participants were contacted from the mailing lists of both universities and were offered to participate in the study in exchange for course credits. Approximately half of the participants were randomly assigned to the “Instructions group” (*N* = 82), and the other half were assigned to the “No instructions group” (*N* = 83). Three participants (one from the Instructions group and two from the No Instructions group) were excluded due to exceptionally low response accuracy (< 70%).

#### Stimuli, design, and procedure

Unless noted otherwise, the procedure of Experiment 2 was similar to that of Experiment 1. The main difference^5^ was how instructions about stimulus-reward contingencies were provided to participants. Participants in the “No Instructions group” did not receive explicit information about the role of the color of the singleton distractor in terms of performance (as in Experiment 1). In contrast, participants in the “Instructions group” were explicitly told that when a high-value singleton color appeared in the display, it would be a “bonus trial,” and they would earn ten times more points than when the low-value singleton distractor was presented, like in Garre-Frutos et al. (2024). To avoid potential issues related to participants not reading the instructions, participants’ knowledge about the instructions was tested using multiple-choice questions at the end of the practice block. Question order and correct response position within each question were randomized. If participants failed to answer correctly, they had to read the instructions again.

In Experiment 2, we also changed the contingency rating test. Data from Experiment 1 suggested that participants might have problems reporting contingency on the raw scale (i.e., the absolute number of points earned with each distractor). Therefore, in Experiment 2, we decided to use a relative scale instead. At the end of the VMAC task, participants were presented with a visual analogue scale (VAS). At each extreme of the VAS, each color singleton was presented with a percentage representing the relative number of points gained with each color. Participants were asked to place a dot on the location on the VAS that best represented the relative number of points gained when each color singleton appeared in the display. Therefore, if a participant moved the dot towards the high-value singleton color, the percentage for each singleton was updated accordingly (e.g., 70% points gained with the high-value singleton and 30% with a low-value singleton). After giving the contingency rating, participants were asked about their confidence in their answer. Participants were again asked to move one point on the VAS to the left or right on a scale from 0 (“not confident”) to 100 (“very confident”). Including this confidence score allows us to assess the validity of our contingency rating through a meaningful correlation with another variable.

#### Data analysis

The analysis in this second experiment differed from Experiment 1 only in the inclusion of Group as an additional predictor and its interaction with the other coefficients in the models described in Experiment 1. The Group predictor was coded using deviation coding to interpret the rest of the coefficients for the overall mean of RTs. As in the previous experiment, for the RT analysis, we excluded incorrect responses (6.54%), outlier responses (RT < 150 ms or RTs > 1,800 ms; 0.45%), and RTs outside each participant’s 2 *SD*s (4.46%).

### Results and discussion

**Fig. 3:**
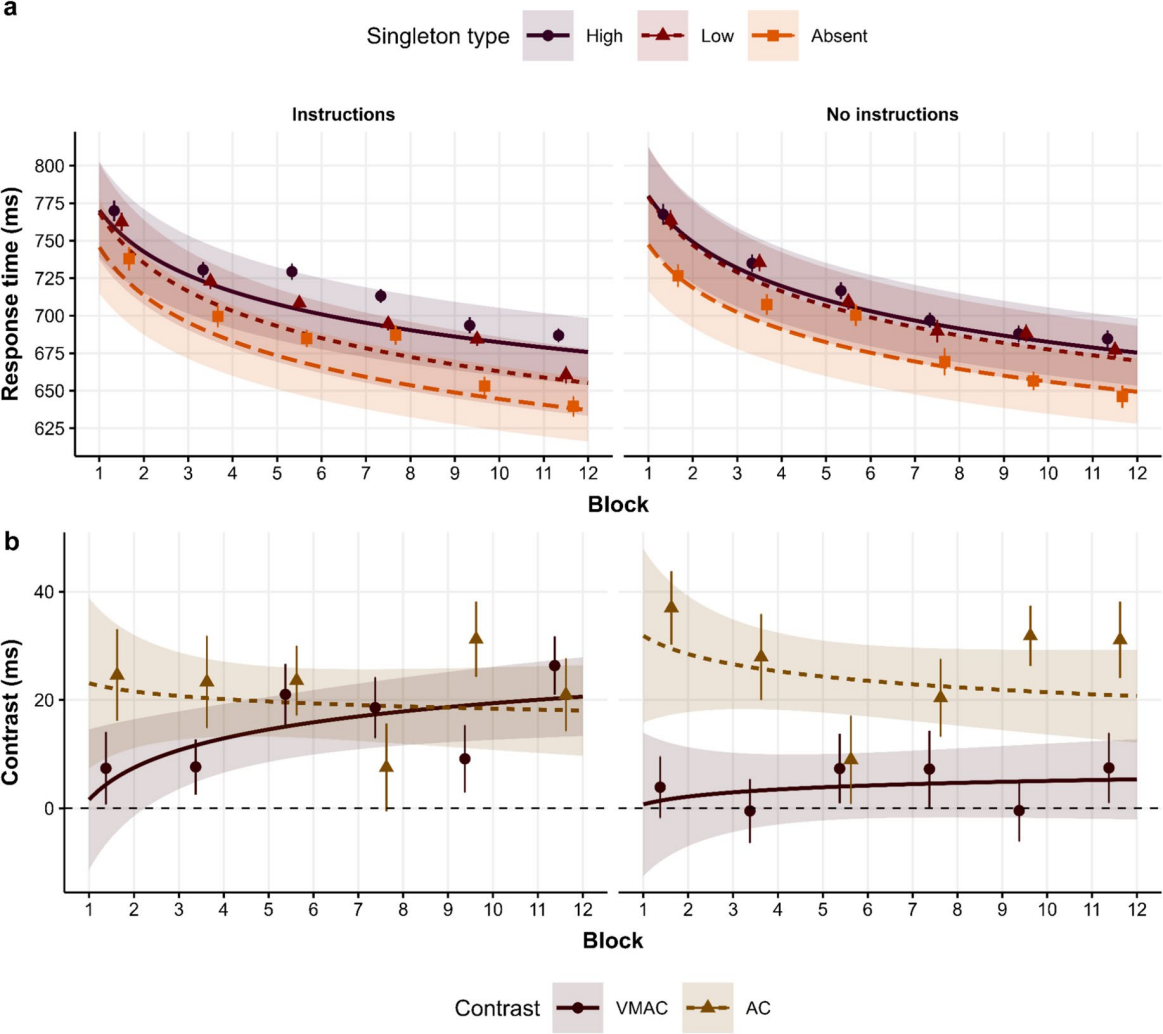
(**a**) Model predictions as a function of singletons across blocks and groups. (**b**) Conditional effects, which represent the conditional mean of the high vs. low (value-modulated attentional capture; VMAC) and low vs. absent (attentional capture; AC) contrasts in the response scale

For RTs (see Table 2), we found a significant AC effect (Instructions: *M*_AC_ = 19.65, 95% CI [13.18, 26.1]; No Instructions: *M*_AC_ = 24.21, 95% CI [17.57, 30.8]) and a significant effect of Block, with RTs decreasing across blocks (Fig. 3a). As for the interaction with the Group predictor, we only found a significant VMAC $\times$ Group interaction, with the Instructions group showing a significant VMAC effect (*M*_VMAC_ = 14.73, 95% CI [8.74, 20.7], *BF*_*10*_* =* 398.66) that was absent in the No instructions group (*M*_VMAC_ = 3.91, 95% CI [−2.18, 10], *BF*_*01*_* =* 3.08) (Fig. 3b).

**Table 2:** Model summaries for the selected models for response times (RTs) and accuracy in Experiment 2

	RTs			Accuracy		
*Predictors*	*Estimates*	*CI*	*p*	*Odds ratios*	*CI*	*p*
(Intercept)	6.519	6.495–6.543	**<0.001**	19.018	17.081–21.175	**<0.001**
VMAC	0.014	0.008–0.020	**<0.001**	1.018	0.933–1.109	0.692
AC	0.032	0.025–0.038	**<0.001**	0.917	0.817–1.030	0.143
Block	−0.042	−0.046 to −0.038	**<0.001**	1.053	0.996–1.113	0.070
Group	−0.012	−0.060–0.036	0.615	1.009	0.816–1.249	0.932
VMAC × Block	0.005	0.001–0.09	**0.020**	1.032	0.947–1.125	0.473
AC × Block	−0.002	−0.007–0.004	0.518	0.936	0.835–1.050	0.259
VMAC × Group	0.016	0.004–0.028	**0.010**	0.848	0.759–1.045	0.061
AC × Group	−0.006	−0.019–0.007	0.375	1.312	1.041–1.654	**0.021**
Block × Group	−0.001	−0.010–0.008	0.513	0.875	0.789–0.971	**0.012**
(VMAC × Block) × Group	0.006	−0.002–0.015	0.157	1.127	0.949–1.339	0.174
(AC × Block) × Group	0.002	−0.009–0.013	0.695	1.016	0.808–1.278	0.892
σ^2^	0.041	3.290				
τ_00_	0.024 _Intercept_	0.382 _Intercept_				
τ_11_	0.001 _VMAC_					
	0.0005 _AC_					
	0.001 _Block_	0.029 _Block_				
ρ_01_	−0.148	0.269				
	0.351					
	−0.279					
ICC	0.377	0.111				
N	162	162				
Observations	41,489	44,129				
Marginal R^2^/Conditional R^2^	0.030/0.396	0.003/0.114				

*Note*. Bold entries denote statistical significance. *p*-values were computed using Satterwhite correction

CI = confidence interval, ICC = intraclass correlation coefficient, τ = random effects, ρ = correlation between random effects

In accuracy, we observed an effect of Block due to increased accuracy across the task. There was also a significant Block × Group interaction, showing that the effect of Block on accuracy was steeper for the No instructions group. We also found a significant AC × Group interaction, where the low-value singleton interfered more with performance in the No Instructions group (*Accuracy*_AC_ **= -**1.02%, 95% CI [−1.7%, −0.02%]) than in the Instructions Group (*Accuracy*_AC_** =** 0.23%, 95% CI [−0.54%, 0.99%]).

**Fig. 4:**
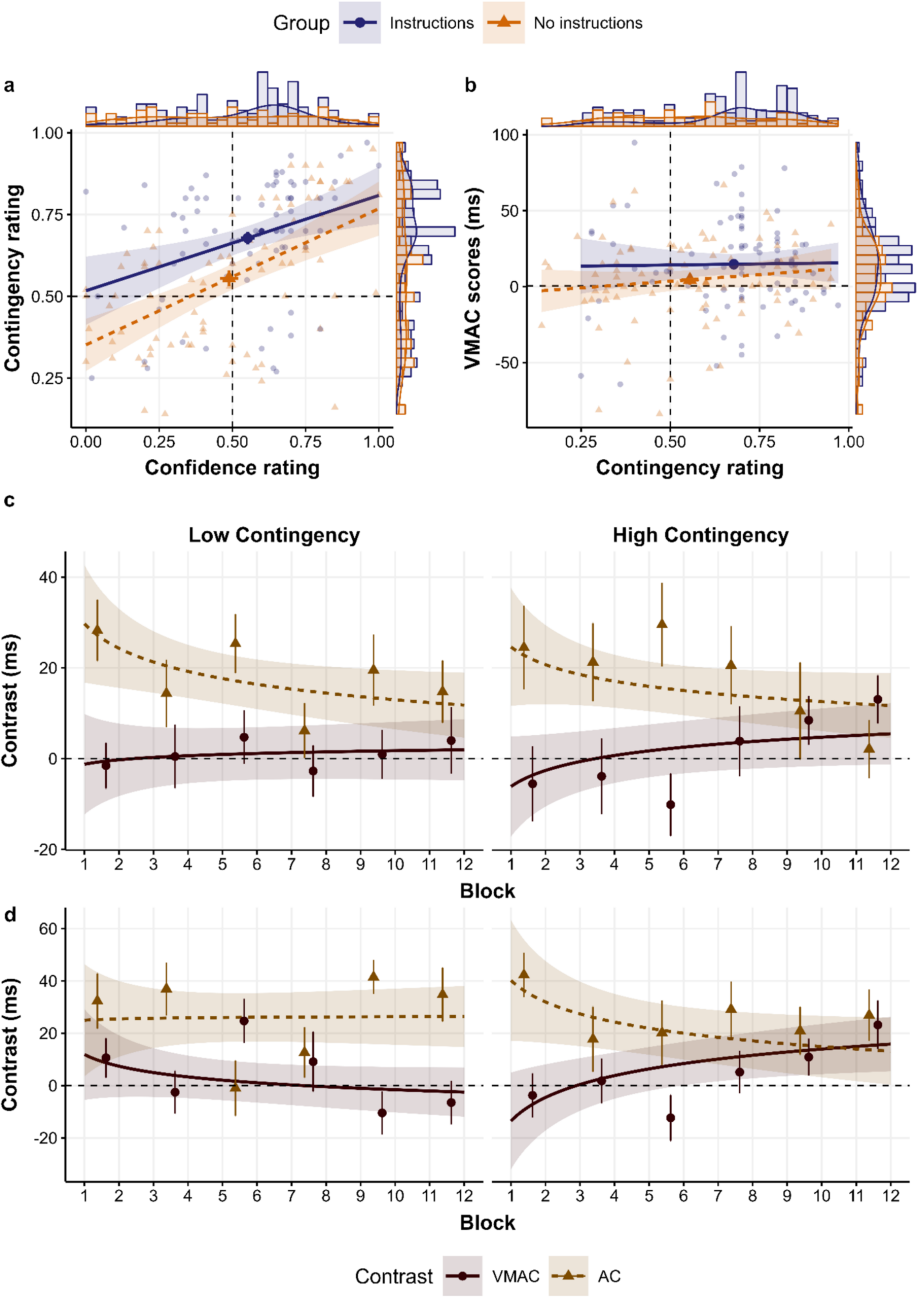
(**a**) Scatterplot for the association between the contingency rating and the confidence rating, where group (Instructions or No instructions) is denoted by different color, shape of the points, and type of line. (**b**) Scatterplot for the association between the value-modulated attentional capture (VMAC) effect and the contingency rating as a function of the group. (**c**, **d**) Conditional VMAC and attentional capture (AC) effect predictions for participants for Experiment 1 (panel c) and the No instructions group in Experiment 2 (panel d) that indicated a high (80th quantile) or low (30th quantile) contingency rating. Raw data represent participants below (*n*_*Experiment 1*_ = 49 and *n*_*Experiment 2*_ = 45) or above (*n*_*Experiment 1*_ = 33 and *n*_*Experiment 2*_ = 38) the 60th quantile of the overall contingency rating distributions of participants in both Experiments. The previous splitting procedure is arbitrary and only for visualization purposes

The contingency rating was positively correlated with confidence ratings in both groups (Instructions: *r* = 0.35, *p* < 0.01, CI 95% [0.14, 0.53]; No instructions: *r* = 0.55, *p* < 0.01, CI 95% [0.38, 0.69]; Fig. 4a), indicating that participants who were more confident in their response showed a better contingency estimation. The Instructions group showed higher contingency ratings (*t*_Welch_(154.86) = 4.023, *p* < 0.01, *M* = 12.28, 95% CI [6.25, 18.31]; Instructions: *M*_Instructions_ = 67.80; No instructions: *M*_No instructions_ = 55.52), but the correlation between VMAC and contingency ratings was not significant in either group (Instructions: *r* = 0.016, *p* = 0.88, CI 95% [−0.20, 0.23], *r*_corrected_ = 0.03; No instructions: *r* = 0.13, *p* = 0.250, CI 95% [−0.09, 0.34], *r*_corrected_ = 0.24; Fig. 4b). A specification curve over this correlation revealed a significant increase when VMAC is computed using data from the later blocks of the task (Figs. S6 and S7, Online Supplementary Material). When contingency rating was added as a predictor, there was a significant three-way interaction between block, VMAC, and contingency awareness for both experiments (Tables S1 and S2, Online Supplementary Material),^6^ showing that uninstructed participants with high awareness showed a progressive increase in VMAC in both experiments (Figs. 4c and d), suggesting that later trials may be mapping a different latent construct.^7^

#### Meta-analysis

To confirm the previous findings, we conducted a meta-analysis of the role of instructions in VMAC. We searched SCOPUS and Web of Science for articles citing the seminal paper by Le Pelley et al. (2015), which also used the one-stage paradigm and coded whether participants received explicit instructions on the contingencies. This resulted in the identification of 59 valid contrasts. In addition, because a previous meta-analysis showed that oculomotor capture tends to have higher effect sizes than RTs (Rusz et al., 2020), we also coded the type of measure used (RTs or eye-tracking) to control for possible differences in sensitivity. Finally, because the number of training trials is critical to the VMAC effect and its relationship to awareness, we also included the length of training as a moderator. To improve the comparability of the magnitude of VMAC with that of the present study, we centered the predictor Training Length on the number of training trials in our experiments. We provide detailed explanations of the study selection criteria, effect size computation, and moderator coding in the Online Supplementary Material.

**Fig. 5:**
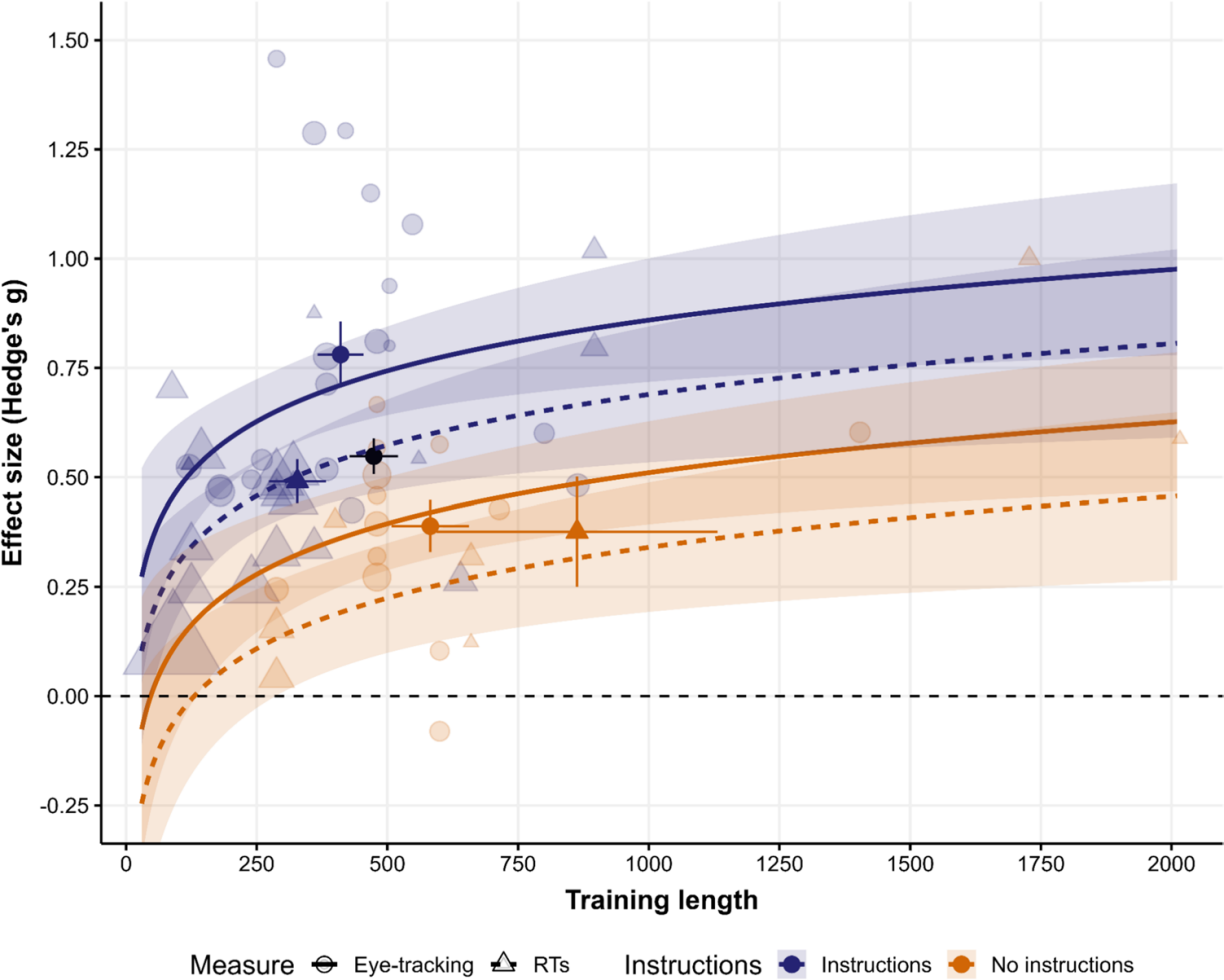
Meta-regression model of the value-modulated attentional capture (VMAC) effect including the number of training trials, type of measure included (response times (RTs) or eye tracking), and whether participants were instructed or not about the stimulus-reward contingencies as moderators. Translucid dots indicate individual effect sizes, and the size of the dot reflects the sample size of a given study. Opaque dots represent the mean effect size and the mean number of training trials as a function of the type of measure (indicated by shape) and instructions (indicated by color). The black dot shows the overall VMAC effect across all studies. Error bars represent SEM

We performed a random effects meta-analysis using the *metafor* R package (Viechtbauer, 2010), showing an overall significant VMAC effect (*g*_*z*_ = 0.51, 95% CI [0.44, 0.58]). The moderator analysis shows that VMAC is significantly influenced by the type of measure (*β*_*Measure*_ = −0.170, *z* = −2.88, *p* =.004), the number of training trials (*β*_*Training length*_ = 0.167, *z* = 3.43, *p* <.001), and, critically, the instructions about the stimulus-reward contingency (*β*_*Instructions*_ = −0.349, *z* = −5.12, *p* <.001).^8^ As displayed in Fig. 5, studies without instructions show lower effect sizes and tend to include a higher number of training trials (*t*_*welch*_(23.438) = −2.73, *p* =.012), suggesting that the impact of instructions is even higher when controlling for differences in training length. Then, we focused on studies without instructions. We fitted the same model with measure and training length as moderators. The meta-analysis revealed a small but significant VMAC effect (*g*_*z*_ = 0.17, 95% CI [0.04, 0.31]),^9^ with only training length influencing effect sizes (*β*_*Training length*_ = 0.262, *z* = 2.96, *p* =.003; *β*_*Measure*_= −0.071, *z* = −0.79, *p* =.423). Additionally, to gather Bayesian evidence, we fitted the same model using the *brms R* package (Bürkner, 2017), showing moderate evidence for the null of no effect in uninstructed studies (*BF*_*01*_ = 3.94).

## General discussion

In two experiments, we investigated the role of awareness on VMAC. In Experiment 1, we observed that when instructions about the stimulus-reward contingency were removed, there was no VMAC effect at the group level. In Experiment 2, we directly manipulated the stimulus-reward contingency instructions in a between-groups design. Critically, only participants who received explicit instructions showed a VMAC effect. Furthermore, although participants without instructions did not show VMAC at the group level, participants who reported higher awareness showed a progressive increase in VMAC through the task, suggesting that awareness of the stimulus-reward relationship may have triggered learning. To confirm our results, we performed a meta-analysis showing that instructions significantly modulate VMAC independently of other moderators. We show a small but significant effect for studies without instructions, while a Bayesian analysis supports the null hypothesis of no effect. Given that the temporal dynamics of VMAC are associated with individual differences in awareness, this small inconsistency may be explained by unaccounted individual differences in contingency awareness at the participant level. However, although this study suggests an absence of VMAC in the one-stage paradigm, particularly in studies measuring RTs, we cannot draw firm conclusions about studies using eye tracking to measure this effect in the two-stage paradigm, as RTs and eye-tracking measures can sometimes lead to different conclusions (Massa et al., 2024; see also DoyLe et al., 2025).

Overall, our results provide a somewhat contradictory picture: when participants know that color informs about the reward schedule, VMAC is significantly magnified, ultimately counterproductive for task goals. Although there is ample evidence that attentional capture by high-value stimuli is automatic, we observed that information provided to participants before learning significantly modulates VMAC. One possible explanation is that instructions elicited an ironic “white bear effect”, i.e., trying to suppress the high-value distractor drives attention towards that distractor (Wegner et al., 1987). Similarly, participants can use their knowledge about the contingency to strategically gather information about the upcoming reward (Mahlberg et al., 2024). Moreover, it is possible that holding the feature-reward association in memory elicits some form of memory-driven attentional capture (OLivers et al., 2006). Under any of these accounts, VMAC should be observed immediately at the beginning of the task. Contrastingly, it is usually the case that VMAC progressively increases with training and it continues to capture attention even if rewards are no longer available (Garre-Frutos et al., 2024, 2025; Watson et al., 2019), which does not fit with any of the previous accounts and may be better explained by a learning-dependent mechanism (see also Failing & Theeuwes, 2017).

VMAC is commonly assumed to reflect increased attention to stimuli that have gained incentive salience through Pavlovian learning (Colaizzi et al., 2020; PelLey et al., 2024). While the consequences of such learning are rather automatic, the learning process may require awareness. In human Pavlovian learning, instructions strongly affect learning (Le Pelley, et al., 2013; Lovibond, 2003; Mertens et al., 2018), and in some instances, such as fear conditioning, instructions alone are sufficient to trigger learning (Cook & Harris, 1937). Some authors have highlighted that expectations and propositional knowledge are critical in human Pavlovian learning (Lovibond & Westbrook, 2024; Mitchell et al., 2009). Consistent with this, contingency awareness is strongly related to learning (Lovibond, 2003; Schmidt & De Houwer, 2012; Weidemann et al., 2016). Even though there is little evidence for unconscious or implicit learning in this field (Lovibond & Shanks, 2002; Mertens & Engelhard, 2020; Shanks, 2010), recent theoretical proposals over the role of learning in attentional control are often referred to as “implicit learning mechanisms” (Anderson et al., 2021; Anderson, 2021; Luck et al., 2021). There is still a debate about whether other “implicit learning mechanisms” are truly implicit (Franco-Martínez et al., 2025; Giménez-Fernández et al., 2020; Meyen et al., 2023; Shanks et al., 2021; Vadillo et al., 2016, 2020b, 2022), but it is possible that implicitness or automaticity is not an all-or-none phenomenon. Psychological processes could share different features associated with automatic and controlled processes, but not necessarily all (Moors & De Houwer, 2006; Ruz & Lupiáñez, 2002). Our results support that awareness of the association between color and reward plays a major modulatory role in the learning process behind VMAC, and even if the attentional capture related to such learning may be conceptualized as automatic by other criteria, we cast doubts about whether its learning process could be considered truly implicit.

Other effects assumed to be implicit, such as visual statistical learning, seem to be less sensible than VMAC to comparable manipulations of awareness (Gao & Theeuwes, 2022) or working memory load (Gao & Theeuwes, 2020; Vicente-Conesa et al., 2024), which are recognized as landmarks of automaticity. Nonetheless, it has been demonstrated that visual statistical learning requires top-down or bottom-up attention to develop (Duncan & Theeuwes, 2020; Duncan et al., 2024; Golan et al., 2024). In the same vein, the mechanism explaining the role of awareness may also relate to how participants direct attention toward the predictive feature. In the one-stage paradigm, the predictive feature is always task irrelevant, which implies that it should receive mostly bottom-up attention (Theeuwes, 1992, 1994), while in the two-stage paradigm, the feature is always relevant during the training stage. One possible explanation for the role of awareness that may clarify discrepancies between the two paradigms is that once participants become aware of the contingency, color is also selectively attended. In other words, learning may depend on the type of attention directed toward the predictive feature rather than on other higher-order processes (Jiang & Chun, 2001; Jiménez & Méndez, 1999; Vadillo et al., 2020a, 2024).

Another (not mutually exclusive) possibility is that participants may learn the same association “explicitly” or “implicitly.” Other studies using different paradigms have found that participants deemed unaware of contingencies using sophisticated signal-detection theory approaches can nevertheless learn Pavlovian attentional biases (Leganes-Fonteneau et al., 2018), and there is evidence that Pavlovian associations can bias attention even if reward feedback is presented below the thresholds of perception (Bourgeois et al., 2016; Seitz et al., 2009), suggesting that learning could occur even when there is no perceptual awareness of the reward. This raises questions about whether “explicit” may qualitatively differ from “implicit” learning (Dayan & Berridge, 2014; PauLi et al., 2019). Recent evidence shows that when feature-reward associations are reversed after a learning stage, VMAC disappears with just a verbal instruction of the reversal in the absence of trial-by-trial feedback (Le et al., 2025), a result often found in human Pavlovian learning (Atlas, 2019). It may be the case that when associations are acquired “explicitly,” information alone can modulate previous learning, while awareness would have little impact on learning when associations are acquired under a different set of conditions.

In conclusion, the present results suggest that awareness of the stimulus-reward relationship may modulate the learning process underlying VMAC. Although VMAC has been considered a highly automatic and implicit process, the concept of automaticity is complex. Once learned, the expression of VMAC may share features of typical automatic processes, but its learning process may not be entirely automatic. Perhaps when participants are instructed (or become aware) about the functional relationship between color and reward, that specific feature (color) is selectively attended, and therefore, learning occurs.

## Supplementary Information

Below is the link to the electronic supplementary material.

Supplementary file1 (DOCX 2459 KB)

## Data Availability

All data related to the present study are publicly available via the Open Science Framework at: https://osf.io/4wdpv/

## Footnotes

1 Reward-driven distraction often receives different names depending on whether the singletons associated with reward are the most salient item in the display (i.e., value “modulates” the attentional capture of an already salient item) or whether all items in the display are equally salient (i.e., value “drives” attentional capture; Anderson et al., 2011b). For the sake of simplicity, we use the term VMAC in both cases.

2 Thanks to Daniel Pearson, we have been able to re-analyze the contingency rating data originally reported by Pearson et al. (2015). As shown in Fig. S2 in the Online Supplementary Material, most participants reported high awareness ratings.

3 Previous studies have employed real (e.g., money) or symbolic (e.g., points) rewards interchangeably. Although one could reasonably expect that this manipulation could affect VMAC, a recent meta-analysis by Rusz et al. (2020) shows that this feature of the task does not significantly moderate the size of the effect. The same conclusion holds even if we restrict the analysis to visual search tasks where reward is task-irrelevant (*Q*(1) = 1.99, *p* = 0.16). We provide a re-analysis of Rusz et al. (2020) in the Online Supplementary Material.

4 We calculated the split-half reliability using a modified version of the *splithalf* R package (Parsons, 2021) that allows stratification. This custom implementation can be found in the following github repository: https://github.com/franfrutos/multi.s

5 In Experiment 2 we employed jspsych (de Leeuw, 2015) instead of OpenSesame. This allowed us to use the virtual-chinrest plug-in (https://www.jspsych.org/7.0/plugins/virtual-chinrest/), which is a direct implementation of the procedure developed by Li et al. (2020), instead of the custom implementation that we created for Experiment 1. We employed the psychophysics plug-in for stimulus presentation (Kuroki, 2021), which has been shown to have a remarkable performance in timing for web-based experiments. In addition, due to the high sample size in this second experiment, participants conducted the task without the experimenter. Nevertheless, instructions for the virtual chinrest were improved to ensure that participants had enough information to understand the task.

6 We also re-analyzed the association between VMAC and the contingency awareness measure in Pearson et al. (2015) using the same approach, and, critically, we found the same result in their data. Namely, VMAC increased through block of trials only for participants able to report the correct contingency between color and reward (Fig. S11, Online Supplementary Material). This further support that awareness is specifically associated with the learning trajectories of VMAC.

7 It is worth noting that in our previous study (Garre-Frutos et al., 2024), a multiverse reliability analysis revealed that the exclusion of the very early trials was detrimental for reliability. A similar result is reported in the Online Supplementary Material of the present study. Therefore, our results suggest that although including more trials may reduce measurement error, the validity of VMAC scores also changes with training, providing further support for a learning-dependent mechanism.

8 We also re-analyzed data from a previous meta-analysis by Rusz et al. (2020) and coded whether participants received explicit instructions about the stimulus-reward contingency. The results show a significant moderator effect (*Q*(1) = 7.43, *p* = 0.006), even when one controls for the sensitivity between RTs and eye-tracking measures (*β*_*Instructions*_ = 0.152, *z* = 2.25, *p* = 0.019).

9 To put the size of this effect in context, to achieve at least 80% statistical power, one would need to recruit at least 261 participants. Only 26 participants would be necessary to achieve the same statistical power for the corresponding effect size in instructed studies (*g*_z_ = 0.58, 95% CI [0.50, 0.65]).

## Code availability

Analysis scripts related to the present study are publicly available via the Open Science Framework at: https://osf.io/4wdpv/.
