# Supplementary material for: Value-modulated attentional capture depends on awareness
Source: Psychon Bull Rev. 2025 Jul 16;32(6):3025–40. doi: 10.3758/s13423-025-02734-1 (PMC12627128; doi:10.3758/s13423-025-02734-1)
Supplement: Supplementary file 1 — Supplementary file1 (DOCX 2459 KB) [file 13423_2025_2734_MOESM1_ESM.docx]

# Supplementary Materials to accompany

# Value-modulated attentional capture depends on awareness

Francisco Garre-Frutos^1,2^, Juan Lupiañez^1,2^, and Miguel A. Vadillo^3^

^1^Mind, Brain, and Behavior Research Center (CIMCYC), University of Granada, Granada, Spain

^2^ Department of Experimental Psychology, University of Granada, Granada, Spain

^3^Department of Basic Psychology, Faculty of Psychology, Autonomous University of Madrid, Madrid, Spain

## Power analysis

As this first experiment is based on Garre-Frutos et al. (2024), we performed a simulation-based power analysis using their results as a reference. We assumed the same model structure and effect sizes observed in Garre-Frutos et al. (2024). Then we tested the significance of the VMAC effect and its interaction with Blocks of trials in 1000 simulations with varying sample sizes (from 20 to 120). Figure S1 shows the proportion of significant results as a function of the statistical test and the number of participants. The power analysis shows that at least 80 participants would be necessary to achieve sufficient power to detect both the VMAC effect (Power = 85.3% 95% CI [83%, 87.4%]) and its interaction with the block of trials (Power = 89.9% 95% CI [87.9%, 91.7%])*^^[[1]](#footnote-0)^^*.


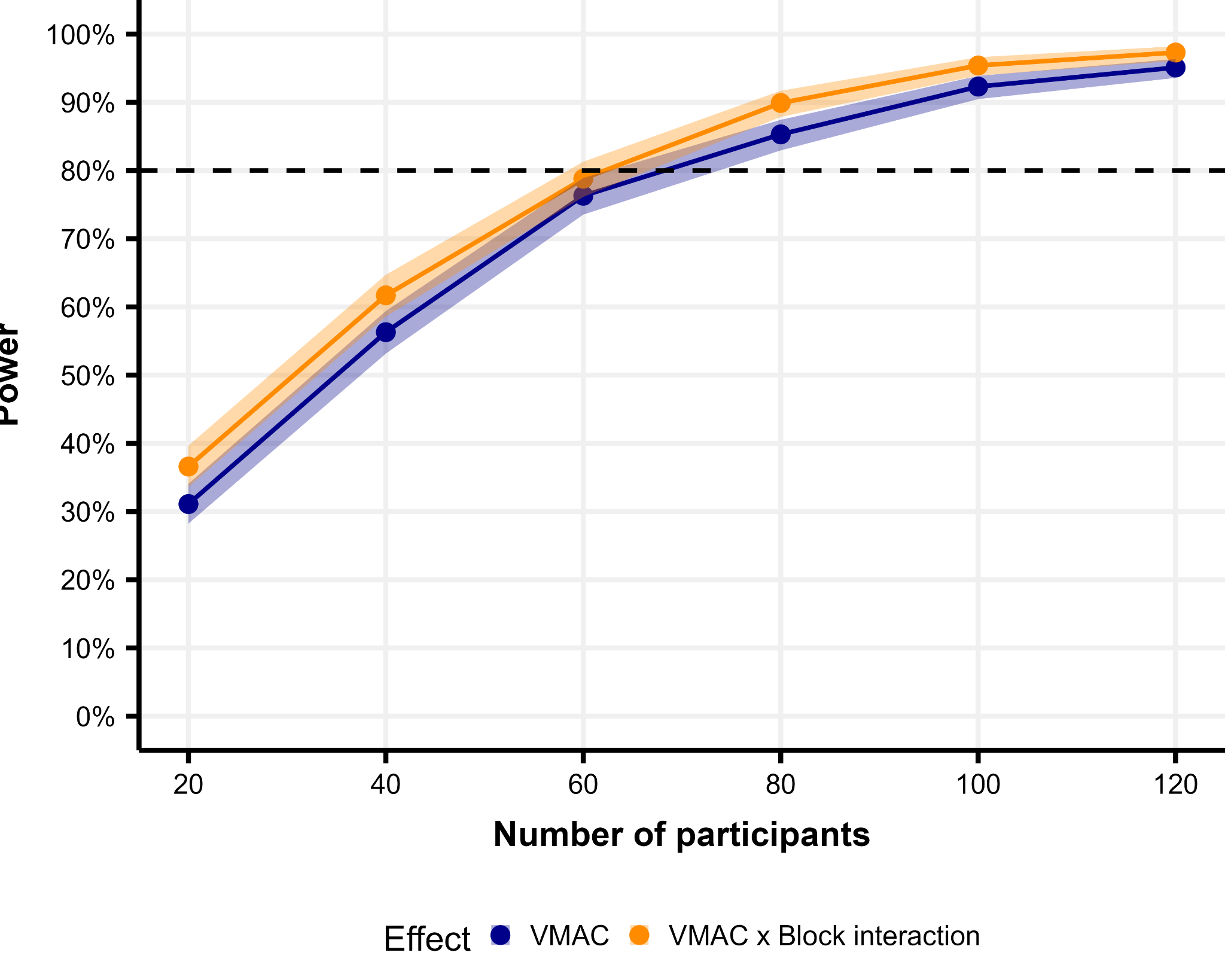


*Figure S1*. Power curve for the VMAC effect and its interaction with the Block predictor. Dots indicated the observed proportion of significant results as a function of the effect and the number of participants employed in the simulation. Shaded segments represent the 95% CI for a binomial proportion.

We did not perform a power analysis for the correlational analysis reported in the main text. Nevertheless, we performed a sensitivity analysis (Lakens, 2022). With the sample size of Experiment 1, we would have 80% statistical power to detect a correlation of .30, and in Experiment 2, we would have power to detect a correlation of at least .31

## Awareness test in Pearson et al. (2015)

Although this was not explicitly assessed, thanks to the first author of Pearson et al. (2015), we had access to the data of the contingency awareness test used in Experiment 2. The contingency assessment designed by Pearson et al. (2015) combined two tests. First, participants had to indicate which of two singleton colours predicted a high or low magnitude reward. Then, participants rated their confidence in their response using a 1-5 Likert scale. The final measure of awareness multiplied the value of the confidence rating by +1 if participants answered the first question correctly or by -1 if participants failed to report the high-value colour. The distribution of contingency ratings across participants is shown in Figure S2. As can be seen, overall contingency awareness was high (*t*(58) = 8.426, *p* < .001; *M_Awareness_* = 2.87, 95% CI[2.19, 3.55]). Based on a binomial test, the overall probability of selecting the correct contingency was well above chance (*probability* = 0.83, *p* < 0.001, CI 95% [0.73, 0.93]), suggesting that most participants were aware of the stimulus-reward contingency.


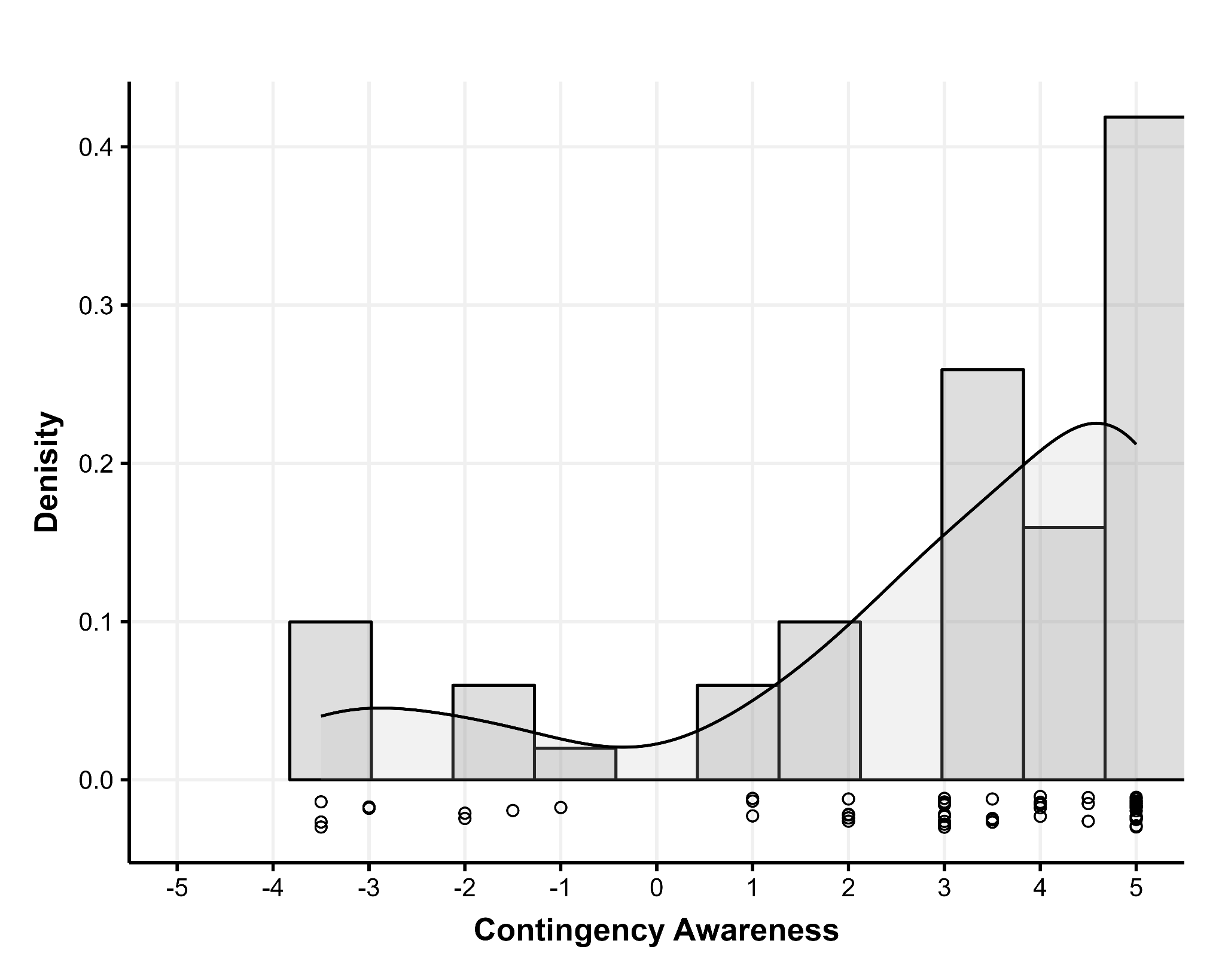
*Figure S2*. Distribution of contingency awareness response on Pearson et al. (2015).

As described above, the awareness test employed by Pearson et al. (2015) was, in fact, a combination of two tests. The logic of combining the two would makes sense if individuals who correctly reported the color-reward contingency and were sure about their response are more likely to present “high” awareness of the correct contingency than individuals who were not sure about their response. In contrast, it is less clear that participants who failed to report the correct contingency would vary on their contingency awareness (capacity to select the correct singleton distractor) as a function of their confidence level. As shown in Figure S3, a logistic regression confirms that the probability of correctly reporting the correct contingency is associated with the confidence rating reported by participants (*β_Confidence_* = 0.79, *z* = 2.53, *p* = .0115), showing that only participants with a low confidence rating tended to report the contingency at chance level. Note that this result is similar to the correlations we report in Experiment 2 for both groups, showing that both measures employed by Pearson et al. (2015) for constructing their contingency rating are related.


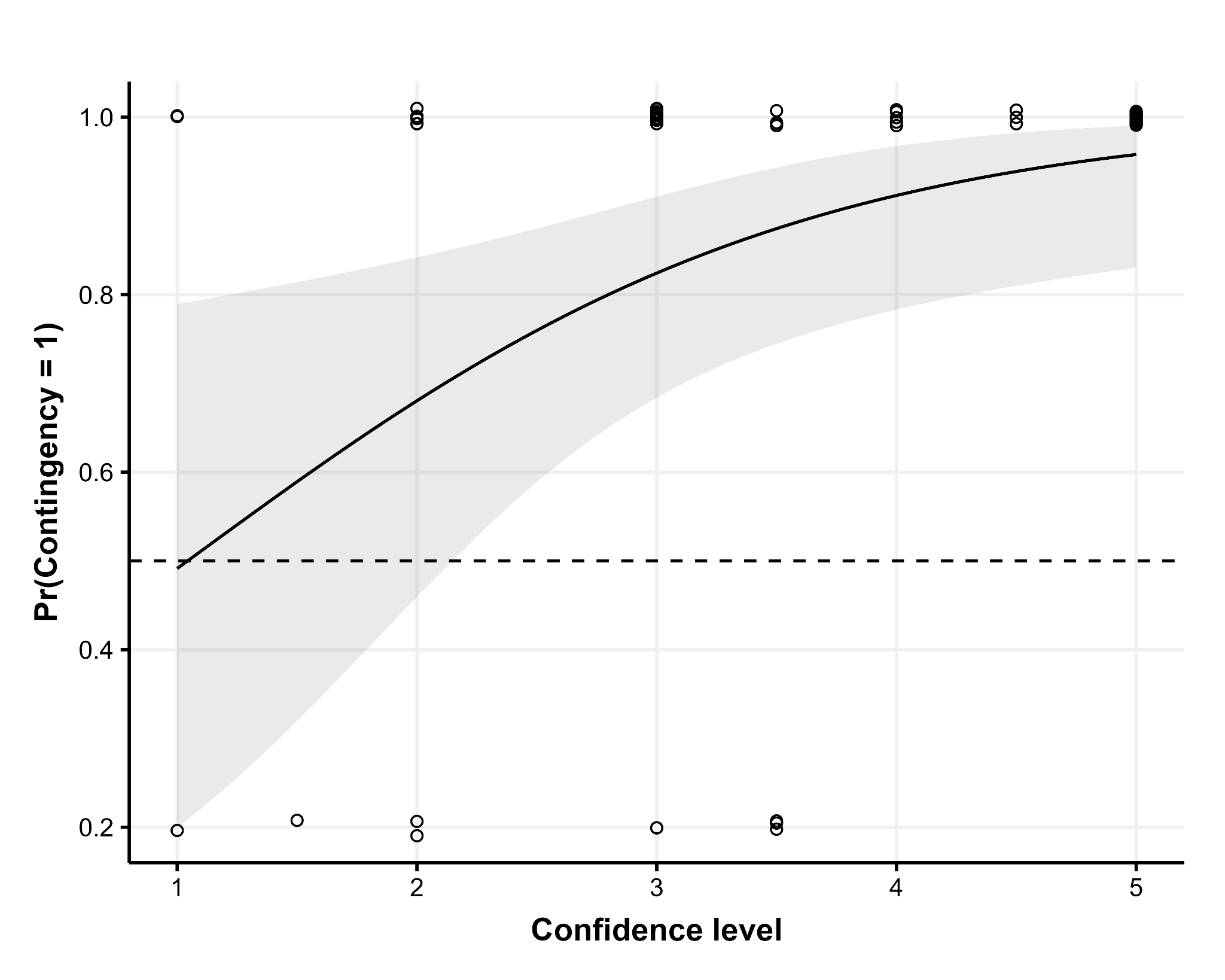


*Figure S3*. Predictions on a logistic regression model predicting the probability of correctly reporting the color-reward contingency and its relationship with the reported confidence in the contingency awareness judgment. The line indicates the expected probability of correct reporting across confidence levels, and the shaded area indicates 95% confidence intervals.

## Re-analyisis of Rusz et al. (2020)

*Type of reward*

The meta-analysis by Rusz et al. (2020) is one of the first meta-analytical reviews on the effect of reward-driven distraction. In that study, a plethora of different moderators on the effect sizes were evaluated. This allowed us to assess to what extent several design decisions made in the present study could have obscured the detection of VMAC. For instance, in the present two experiments, participants were rewarded with symbolic rewards (points), which were not translated to any economic reward. This could potentially affect the effect of reward during the task. Nevertheless, in the meta-analysis of Rusz et al. (2020), the type of reward did not influence the size of reward-driven distraction in any way. Even if we restrict the moderator analysis to visual search tasks, the type of reward employed remains a non-significant moderator (*Q*(1) = 1.99, *p* = 0.16).

*Instructions about the stimulus-reward contingencies*

As in the “Meta-analysis” section of the main text, we also re-analyzed Rusz et al. (2020) to test whether providing instructions about the stimulus-reward contingency significantly moderates the size of the effect. To that aim, we coded the inclusion (or not) of instructions regarding the stimulus-reward contingencies in all the studies selected by Rusz et al. (2020) and included “Instructions” as a moderator. The results show that instructing participants about the stimulus-reward contingencies produces a significant increase in the size of reward-driven distraction across studies (*Q*(1) = 7.43, *p* = 0.006). The same result holds even when controlling for the sensitivity of different measures (*β_Instructions_* = 0.152, *z* = 2.25, *p* = 0.019).

## Reliability multiverse analysis for Experiments 1 and 2

Here, we present a multiverse reliability analysis exploring the reliability of our measures under a plausible range of data preprocessing specifications (Parsons, 2022). Similar to Garre-Frutos et al. (2024), we orthogonally manipulated the following factors:

- Relative filter for RTs: none, 2 SDs, 2.5 SDs, or 3 SDs.
- Fixed filter for RTs: none or (RT > 150 and RT < 1800),
- Averaging method: mean or median.
- Log-transform RTs: yes or no.
- Filter the first two trials of each block: yes or no.
- Number of blocks used to calculate the effect: 6 or 12.

The combination of the previous factors gives rise to 128 possible data preprocessing specifications. For every specification, we calculated random permutated split-half reliability using 5000 random permutations (Parsons, 2021). Then, we corrected the reliability estimates using the Spearman-Brown formula. Figures S2, S3, and S4 (top row) show the mean Spearman-Brown estimates across permutations and the 95% bootstrapped CIs ordered as a function of reliability. In contrast, the bottom row shows the corresponding specification for each reliability estimate in the top row.


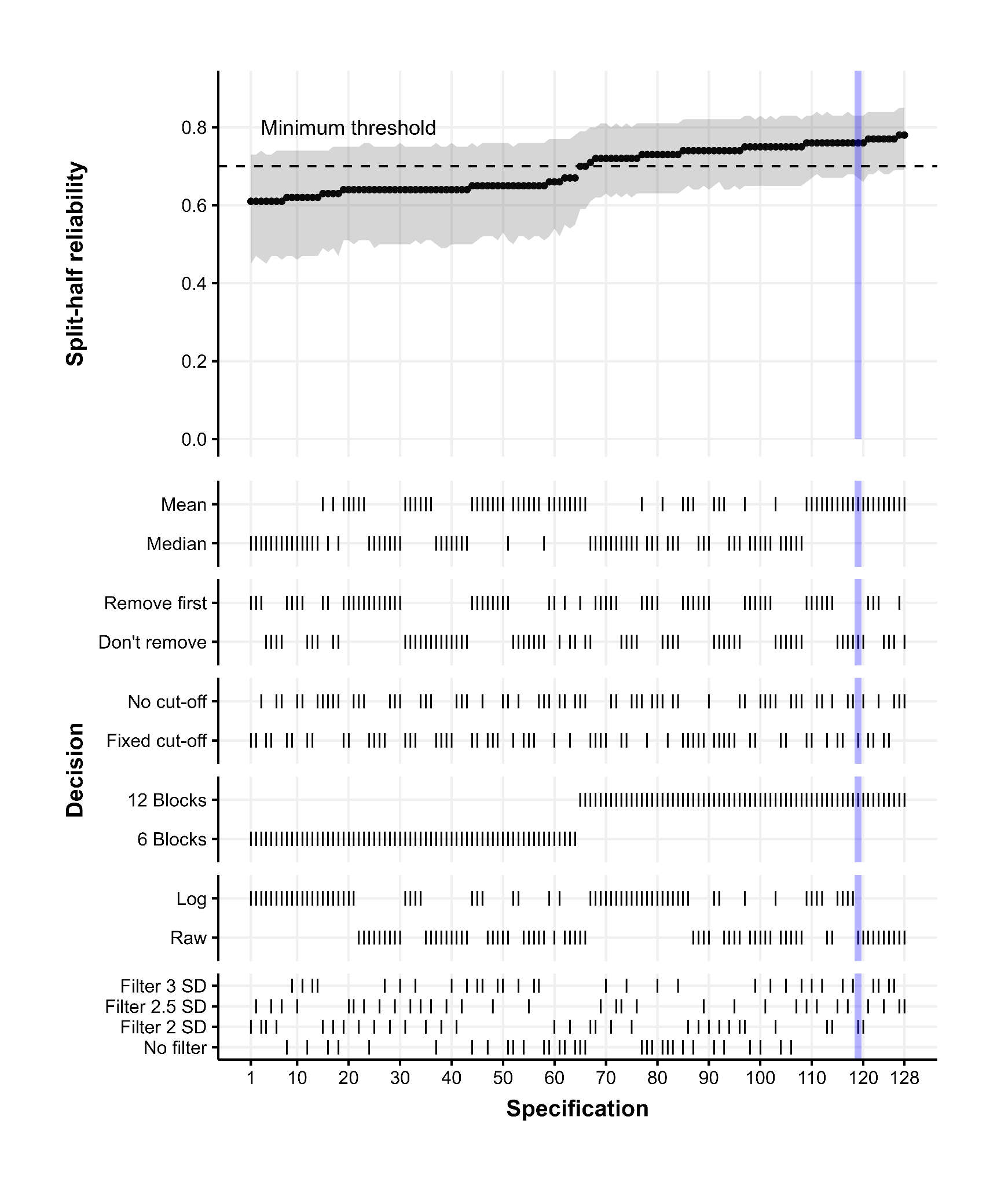


*Figure S4.* In the top panel, each dot represents a Spearman-Brown reliability estimate in Experiment 1, and shaded areas represent 95% CI. Regarding the bottom panel, the different combinations of specifications are signaled with a vertical line. The line in the top panel highlights 0.7 as the minimum threshold for studies on individual differences, and the vertical shaded segment represents the specification used in the main analysis.
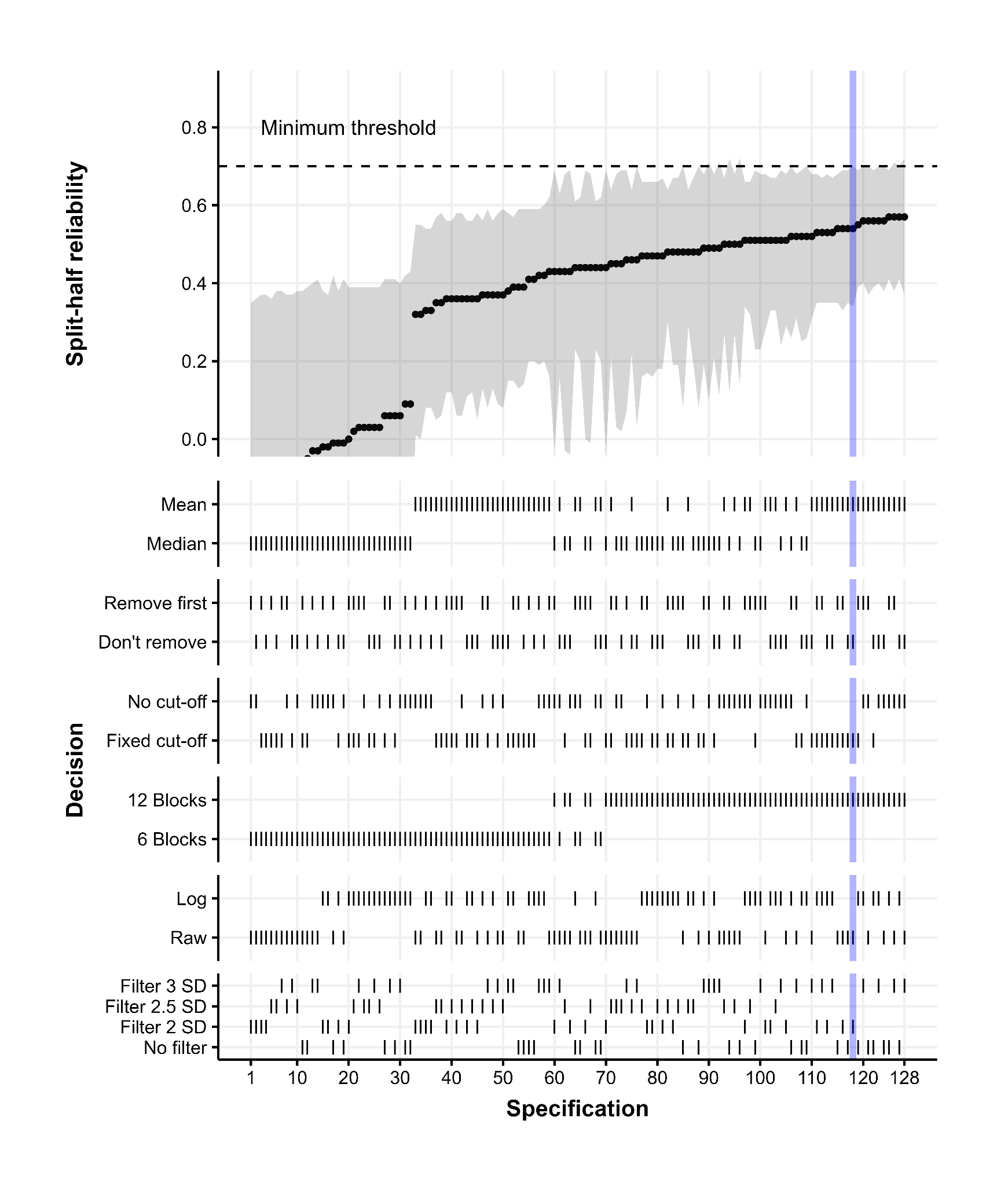


*Figure S5.* In the top panel, each dot represents a Spearman-Brown reliability estimate for the Instructions group in Experiment 2. Shaded areas represent 95% CI. The bottom panel depicts the different combinations of specifications signaled with a vertical line. The line in the top panel highlights 0.7 as the minimum threshold for studies on individual differences, and the vertical shaded segment represents the specification used in the main analysis.


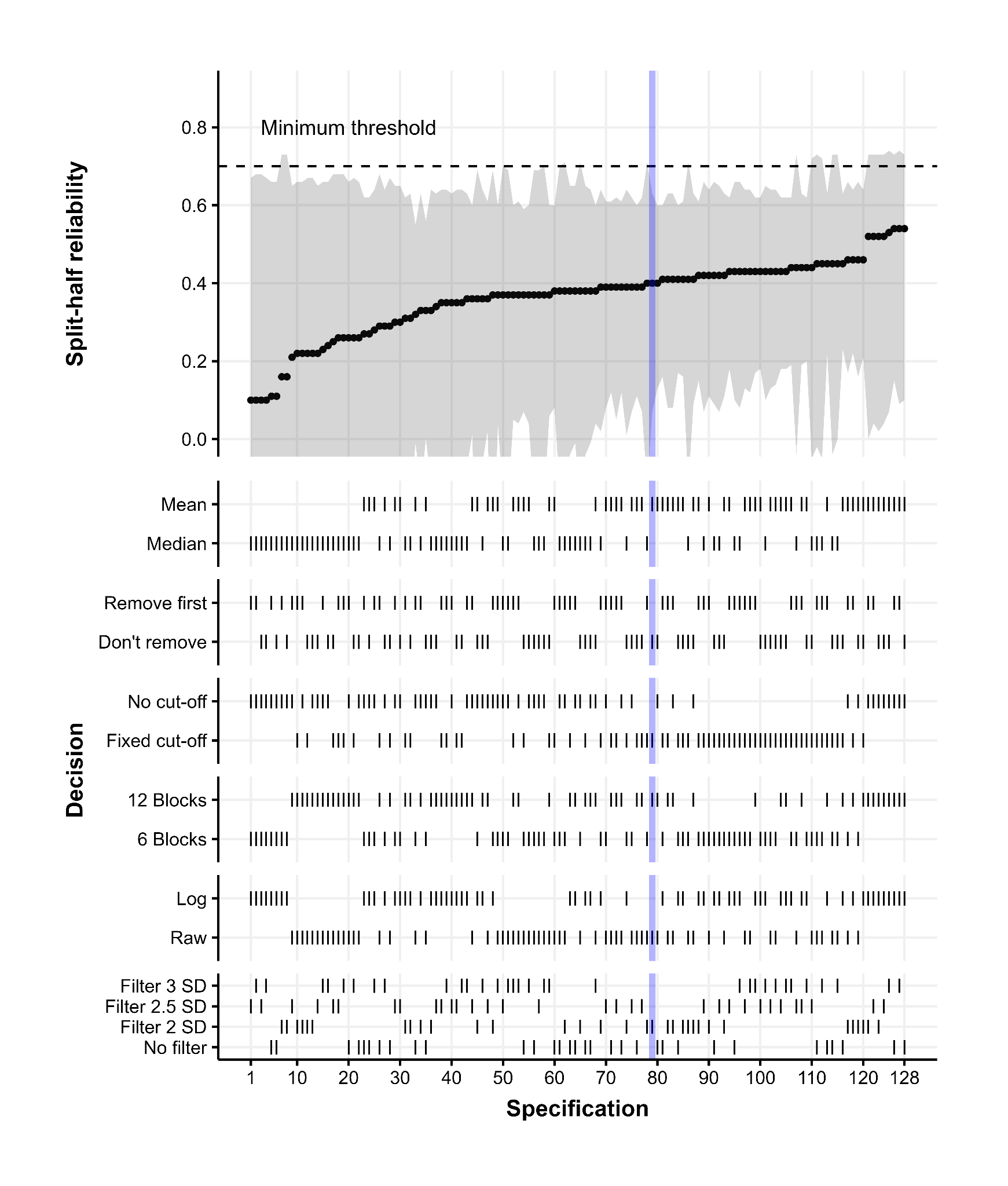


*Figure S6.* In the top panel, each dot represents a Spearman-Brown reliability estimate for the No Instructions group in Experiment 2. Shaded areas represent 95% CI. The bottom panel depicts the different combinations of specifications signaled with a vertical line. The line in the top panel highlights 0.7 as the minimum threshold for studies on individual differences, and the vertical shaded segment represents the specification used in the main analysis.

In Experiment 1 (Figure S2), the median reliability is *r*_sb_ = 0.67, 95% CI [0.55, 0.78], and the range of reliabilities is [0.61, 0.78], with 48.43% of the estimates above the usual recommended minimum threshold for individual difference research (0.7, following Nunnally, 1978). For Experiment 2, we show the reliability of our measures in Figures S3 (Instruction group: median of *r*_sb_ = 0.44, 95% CI [0.23, 0.61], range of [-0.14, 0.57]) and S4 (No instructions group: median of *r*_sb_ = 0.38, 95% CI [-0.1, 0.64], range of [0.1, 0.54]). As can be seen, in general, reliability in either group of Experiment 2 is lower than that observed in Experiment 1. This may be related to the experimenter's presence via the online meeting in Experiment 1 compared to Experiment 2 (the main difference in both experiments). Nevertheless, the reliability of the particular specification employed in the analysis of Experiment 2 is quite high, considering the overall range of reliabilities.

## Specification curve analysis for the correlation between VMAC scores and contingency rating

Given the null correlation between the VMAC scores in Experiment 2 and the measure of contingency is highly correlated with the confidence rating, we decided to examine the extent to which this correlation could be attenuated by measurement error. To do this, we used the data sets generated for the previous multiverse analysis and computed the correlation between the VMAC scores and the contingency rating for each group. If the observed correlation is attenuated by measurement error, it should increase as a function of the reliability of the VMAC scores, especially for the No Instructions group (since we expect the contingency rating to measure awareness only for participants who did not receive explicit instructions).


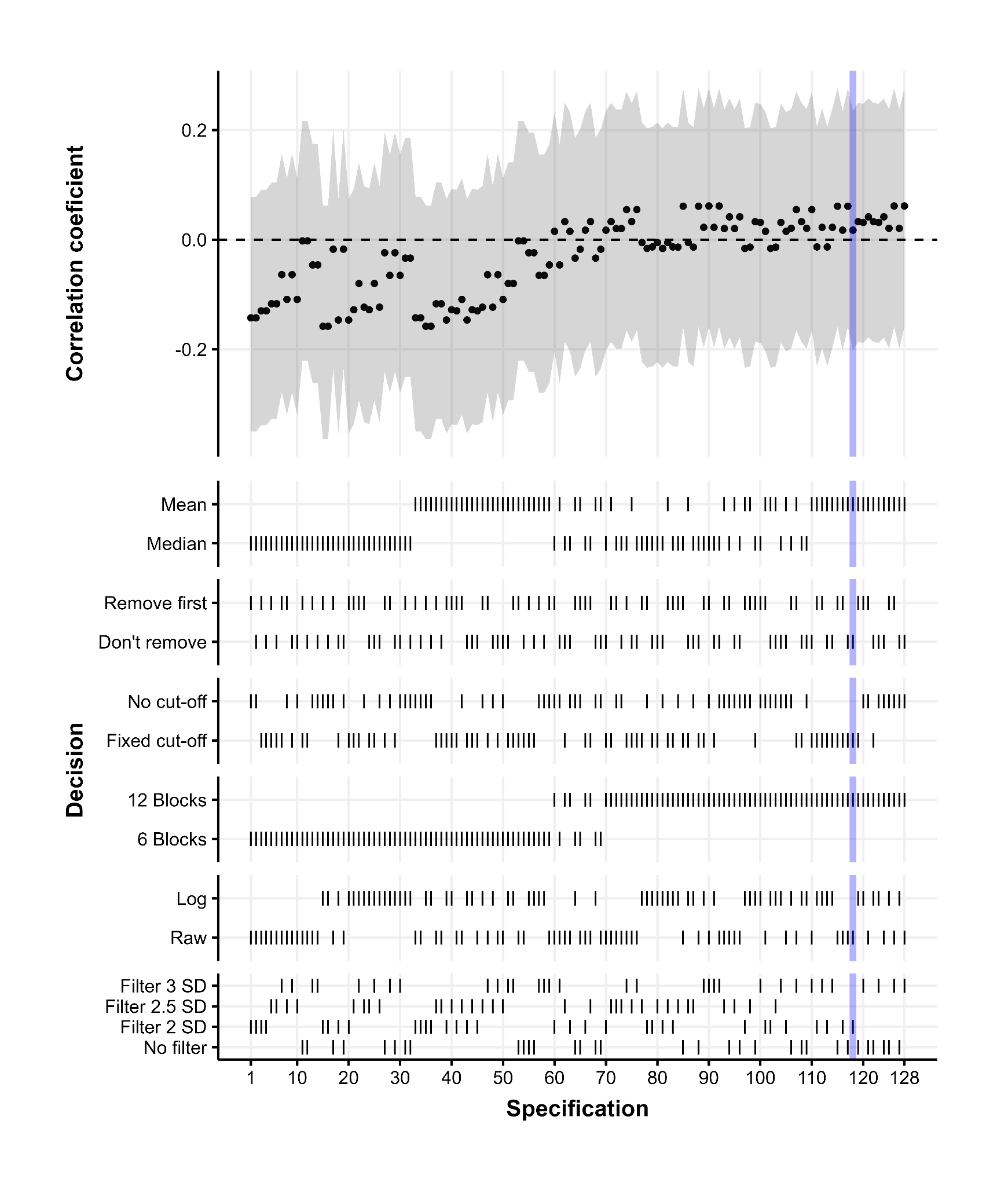


*Figure S7.* In the top panel, each dot represents the correlation between VMAC and the contingency rating for the Instructions group ordered by the reliabilities of different data preprocessing specifications. Shaded areas represent 95% CI for the same correlation. The bottom panel illustrates different specifications, where the vertical line signals the possible combinations of factors employed for each specification.


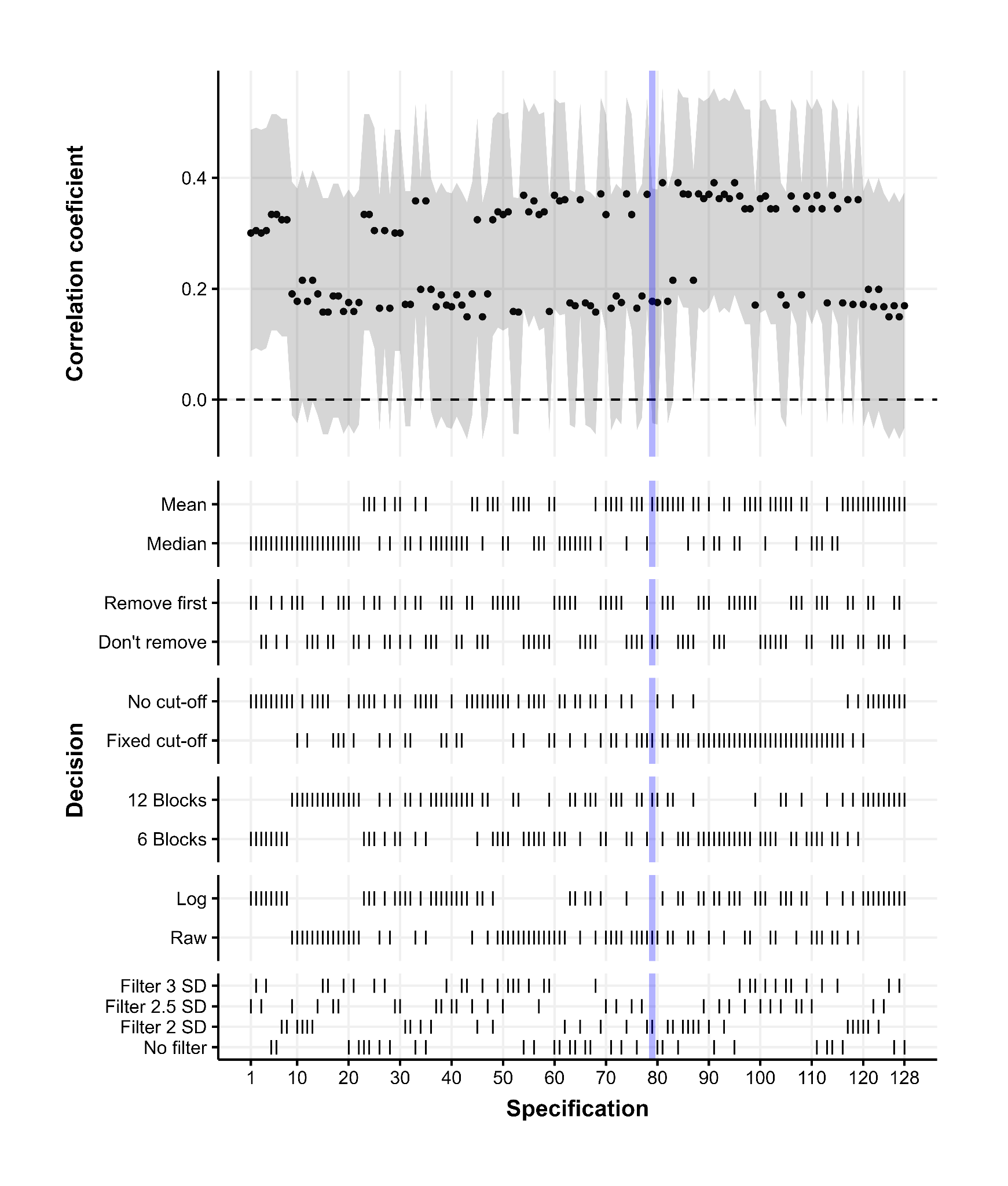


*Figure S8.* In the top panel, each dot represents the correlation between VMAC and the contingency rating for the no-instructions group ordered by the reliabilities of different data preprocessing specifications. Shaded areas represent 95% CI for the same correlation. The bottom panel illustrates different specifications, where the vertical line signals the possible combinations of factors employed for each specification.

Figures S7 and S8 show the correlation between VMAC and contingency ratings as a function of the reliability of each specification for both groups, respectively. In the Instructions group, there is a clear pattern where specifications with lower reliability tend to show a negative correlation. Additionally, these specifications often use the last half of the task to calculate the VMAC effect. This pattern could resemble regression to the mean due to high measurement error (Shanks, 2017). Specifically, participants with extreme VMAC scores may have less extreme contingency ratings, creating a spurious negative association. In contrast, in the No Instructions group, the correlation coefficient systematically increases for every specification when VMAC scores are computed using the last six blocks of trials. This pattern is more evident in Figure S7.


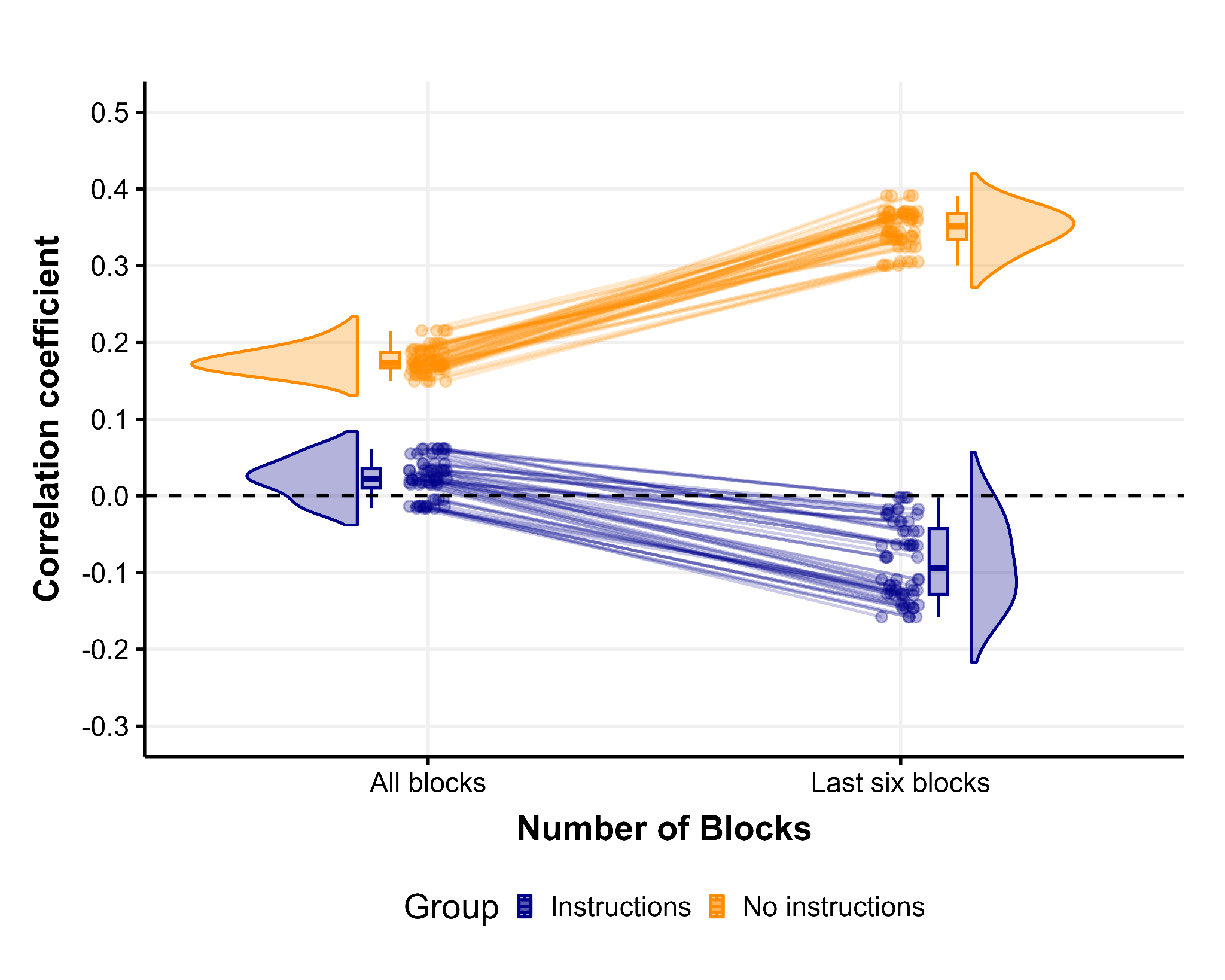
*Figure S9*. Spearman Correlation between VMAC effect and the contingency rating in Experiment 2 across different sets of data preprocessing specifications. The x-axis subsets these correlations as a function of the number of blocks employed to calculate the VMAC effect.

The previous analysis suggests that the underlying construct might vary depending on whether all trials or only the last half of the task are included in the calculation of the VMAC effect. In the No Instructions group, where this relationship is stronger, later trials may reflect that some participants become spontaneously aware of the contingency and start to learn later. Consequently, individual differences in contingency estimation could reliably predict VMAC scores. This means the underlying correlation may consistently change because later trials may better capture true individual differences in the learning process of VMAC. This temporal dependency suggests that the temporal dynamics of the VMAC effect should vary as a function of the contingency rating test.


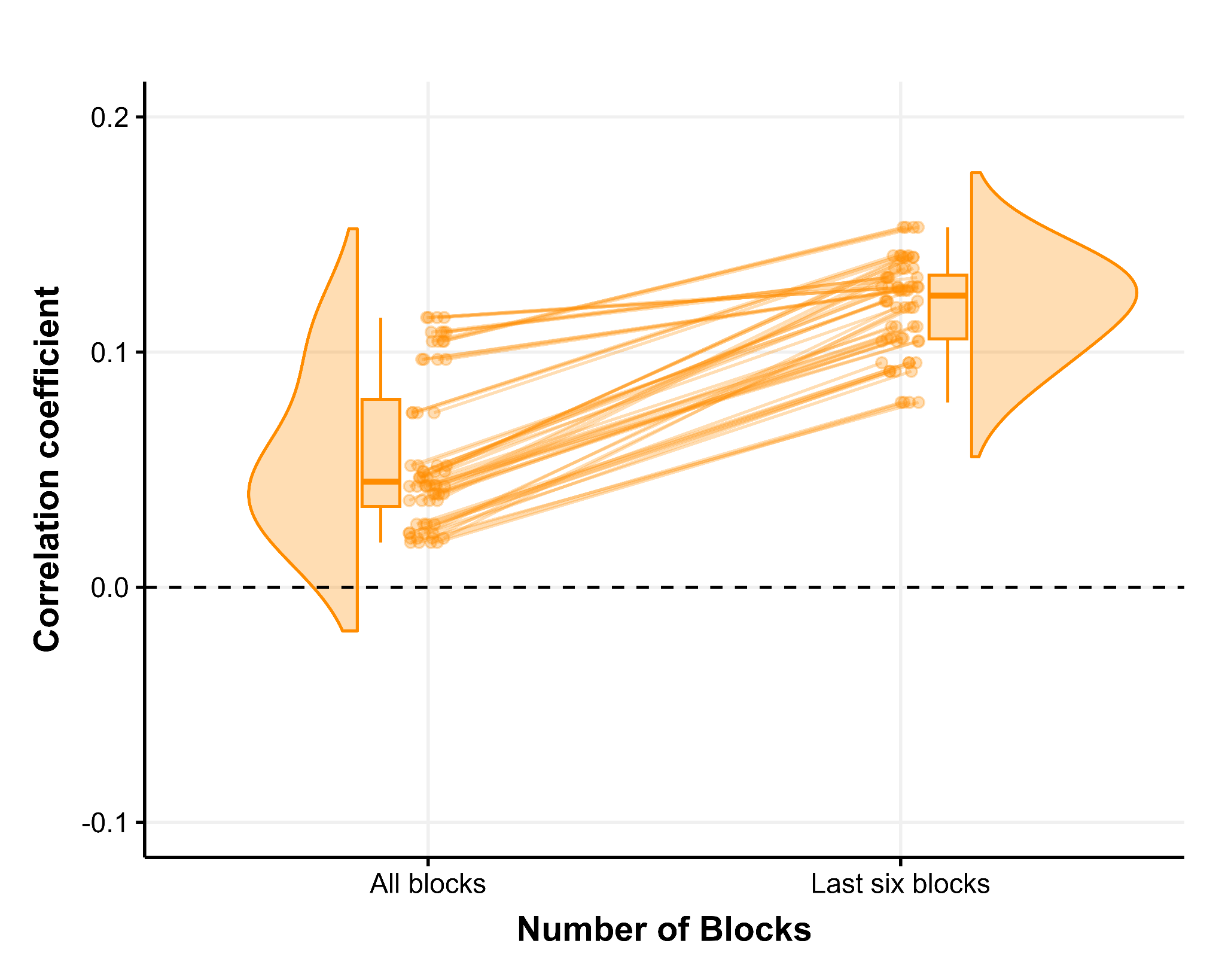
*Figure S10*. Spearman Correlation between VMAC effect and the contingency rating in Experiment 1 across different sets of data preprocessing specifications. The x-axis subsets these correlations as a function of the number of blocks employed to calculate the VMAC effect.

To investigate this possibility, we fit a linear mixed model following the same structure used in the analysis of Experiment 2 for the No Instructions group, adding the scaled contingency rating as a predictor. Table S1 (left) shows the model coefficients. We observed a significant effect of the contingency predictor, where participants with a higher contingency rating had faster RTs than participants with a lower contingency rating. Critically, there was only a significant VMAC x Block x Contingency interaction, which is visualized in Figure 4d of the main text. Table S1 (right) presents the accuracy analysis, also including the contingency rating as a predictor. This analysis reveals no significant effect or interaction with the Contingency predictor, suggesting that the previous analysis does not reflect a speed-accuracy tradeoff.

**Table S1**

*Model summaries for the selected models for RTs and accuracy in experiment 2.*

|  | **RTs** | | | **Accuracy** | | |
| --- | --- | --- | --- | --- | --- | --- |
| *Predictors* | *Estimates* | *CI* | *p* | *Odds Ratios* | *CI* | *p* |
| (Intercept) | 6.525 | 6.495 – 6.555 | **<0.001** | 18.672 | 15.894 – 21.935 | **<0.001** |
| VMAC | 0.006 | -0.003 – 0.014 | 0.177 | 1.105 | 0.970 – 1.246 | 0.105 |
| AC | 0.035 | 0.024 – 0.045 | **<0.001** | 0.800 | 0.678 – 0.944 | **0.008** |
| Block | -0.041 | -0.048 – -0.035 | **<0.001** | 1.106 | 1.010 – 1.197 | **0.012** |
| Contingency | -0.039 | -0.069 – -0.009 | **0.011** | 1.004 | 0.858 – 1.175 | 0.769 |
| VMAC × Block | 0.002 | -0.004 – 0.008 | 0.537 | 0.929 | 0.789 – 1.095 | 0.678 |
| AC × Block | -0.003 | -0.011 – 0.005 | 0.488 | 0.929 | 0.789 – 1.095 | 0.381 |
| VMAC × Contingency | 0.005 | -0.004 – 0.013 | 0.274 | 0.913 | 0.813 – 1.025 | 0.123 |
| AC × Contingency | -0.003 | -0.013 – 0.008 | 0.618 | 0.998 | 0.951 – 1.171 | 0.983 |
| Block × Contingency | 0.005 | -0.002 – 0.012 | 0.130 | 0.935 | 0.970 – 1.005 | 0.0068 |
| (VMAC × Block) × Contingency | 0.010 | 0.003 – 0.016 | **0.003** | 0.983 | 0.875 – 1.103 | 0.769 |
| (AC × Block) × Contingency | -0.007 | -0.015 – 0.001 | 0.109 | 1.025 | 0.876 – 1.200 | 0.759 |
| **Random Effects** | | | | | | |
| σ^2^ | 0.043 | | | 3.290 | | |
| τ_00_ | 0.016 _Intercept_ | | | 0.431 _Intercept_ | | |
| τ_11_ | 0.001 _VMAC_ | | |  | | |
|  | 0.001 _AC_ | | |  | | |
|  | 0.001 _Block_ | | | 0.032 _Block_ | | |
| ρ_01_ | -0.038 | | | 0.381 | | |
|  | 0.146 | | |  | | |
|  | -0.224 | | |  | | |
| ICC | 0.319 | | | 0.116 | | |
| N | 81 | | | 81 | | |
| Observations | 20,644 | | | 21,997 | | |
| Marginal R^2^ / Conditional R^2^ | 0.052 / 0.355 | | | 0.006 / 0.121 | | |

*Note*. Bold entries denote statistical significance. *p*-values were computed using Satterwhite correction. CI = Confidence interval; ICC = Intraclass correlation coefficient. τ = Random effects, ρ = correlation between random effects.

Due to the interesting insights about the temporal dynamics of the VMAC effect in Experiment 2 for participants who did not receive information about the color-reward contingency, we checked if the same pattern could be observed in Experiment 1. As in the previous analysis, Figure S8 shows that the correlation between the VMAC effect and the contingency rating increases when VMAC scores are computed in the last half of the task compared to the whole task. Therefore, we fit the same models described in the main text for Experiment 1, adding the scaled Contingency rating as a predictor. Critically, in the RTs analysis, the contingency rating predictor only significantly interacted with the VMAC x Block interaction, and there was no significant effect nor interaction with the contingency rating in the accuracy analysis (Table S2). In Figure 4c of the main text, we visualized this interaction. This analysis further suggests that a high contingency rating is associated with an increased VMAC effect later in the task and that the true correlation between our measures of awareness and the VMAC effect seems to be time-dependent, as has been proposed in other learning-dependent attentional effects (Meyen et al., 2023; Vadillo et al., 2022).

**Table S2**

*Model summaries for the selected models for RTs and accuracy in experiment 1.*

|  | **RTs** | | | **Accuracy** | | |
| --- | --- | --- | --- | --- | --- | --- |
| *Predictors* | *Estimates* | *CI* | *p* | *Odds Ratios* | *CI* | *p* |
| (Intercept) | 6.462 | 6.435 – 6.488 | **<0.001** | 20.343 | 16.070 – 25.754 | **<0.001** |
| VMAC | 0.002 | -0.006 – 0.011 | 0.590 | 1.025 | 0.787 – 1.335 | 0.855 |
| AC | 0.025 | 0.017 – 0.033 | **<0.001** | 0.893 | 0.624 – 1.278 | 0.538 |
| Block | -0.036 | -0.042 – -0.029 | **<0.001** | 1.035 | 1.009 – 1.062 | **0.009** |
| Contingency | -0.003 | -0.032 – 0.026 | 0.765 | 0.963 | 0.771 – 1.202 | 0.736 |
| VMAC × Block | 0.003 | -0.002 – 0.008 | 0.226 | 1.003 | 0.966 – 1.041 | 0.891 |
| AC × Block | -0.006 | -0.012 – 0.001 | 0.099 | 1.003 | 0.953 – 1.055 | 0.913 |
| VMAC × Contingency | 0.003 | -0.006 – 0.011 | 0.537 | 0.870 | 0.664 – 1.139 | 0.310 |
| AC × Contingency | -0.003 | -0.012 – 0.004 | 0.320 | 1.321 | 0.928 – 1.879 | 0.122 |
| Block × Contingency | 0.000 | -0.006 – 0.007 | 0.878 | 1.007 | 0.984 – 1.031 | 0.557 |
| (VMAC × Block) × Contingency | 0.006 | 0.001 – 0.011 | **0.020** | 0.995 | 0.959 – 1.035 | 0.854 |
| (AC × Block) × Contingency | 0.003 | -0.003 – 0.010 | 0.304 | 0.972 | 0.923 – 1.024 | 0.281 |
| **Random Effects** | | | | | | |
| σ^2^ | 0.031 | | | 3.290 | | |
| τ_00_ | 0.015 _Intercept_ | | | 0.632 _Intercept_ | | |
| τ_11_ | 0.001 _VMAC_ | | |  | | |
|  | 0.0001 _AC_ | | |  | | |
|  | 0.001 _Block_ | | | 0.003 _Block_ | | |
| ρ_01_ | 0.025 | | | -0.393 | | |
|  | 0.346 | | |  | | |
|  | -0.272 | | |  | | |
| ICC | 0.346 | | | 0.148 | | |
| N | 82 | | | 82 | | |
| Observations | 21,445 | | | 21,554 | | |
| Marginal R^2^ / Conditional R^2^ | 0.030 / 0.365 | | | 0.006 / 0.153 | | |

*Note*. Bold entries denote statistical significance. *p*-values were computed using Satterwhite correction. CI = Confidence interval; ICC = Intraclass correlation coefficient. τ = Random effects, ρ = correlation between random effects.

## Re-analysis of the association between contingency awareness and VMAC in Pearson et al. (2015)

Based on the re-analysis provided in the previous section, where we show that the learning process of VMAC is associated with our awareness measure, we run a similar re-analysis on the data collected by Pearson et al. (2015), which also evaluated the association between VMAC and an awareness test with an eye-tracking version of the task. Specifically, we fitted a GLMM on the probability of fixating a singleton distractor, including as factors the type of Singleton (high-value, low-value, and absent distractor), the Block of trials (1 - 20), and the score from the awareness test. As explained in the data analysis section of Experiments 1 and 2, we set the hypothesis matrix of the Singleton predictor to have a VMAC contrast (high-value vs. low-value) and an AC contrast (low-value vs. absent distractor), and the Block and Awareness predictors were mean-centered and scaled.

**Table S3**

*Model summaries for re-analysis of Pearson et al. (2015) with awareness scores.*

|  |  | | |
| --- | --- | --- | --- |
| *Predictors* | *Odds Ratios* | *CI* | *p* |
| (Intercept) | 0.08 | 0.06 – 0.09 | **<0.001** |
| VMAC | 1.01 | 0.93 – 1.09 | 0.894 |
| AC | 1.52 | 1.23 – 1.88 | **<0.001** |
| Block | 4.21 | 3.00 – 5.90 | **<0.001** |
| Contingency | 1.01 | 0.82 – 1.24 | 0.915 |
| VMAC × Block | 1.29 | 1.19 – 1.40 | **<0.001** |
| AC × Block | 0.92 | 0.77 – 1.09 | 0.330 |
| VMAC × Contingency | 1.01 | 0.93 – 1.09 | 0.871 |
| AC × Contingency | 1.19 | 0.96 – 1.46 | 0.110 |
| Block × Contingency | 0.87 | 0.64 – 1.19 | 0.392 |
| (VMAC × Block) × Contingency | 1.19 | 1.09 – 1.29 | **<0.001** |
| (AC × Block) × Contingency | 0.94 | 0.78 – 1.12 | 0.471 |
| **Random Effects** | | | |
| σ^2^ | 3.29 | | |
| τ_00_ | 0.56 _Intercept_ | | |
| τ_11_ | 0.03 _Block_ | | |
| τ_11_ | 0.53 _VMAC_ | | |
| τ_11_ | 0.90 _AC_ | | |
| ρ_01_ | 0.08 | | |
|  | -0.07 | | |
|  | 0.06 | | |
| ICC | 0.21 | | |
| N | 59 | | |
| Observations | 3523 | | |
| Marginal R^2^ / Conditional R^2^ | 0.136 / 0.314 | | |

*Note*. Bold entries denote statistical significance. *p*-values were computed using Satterwhite correction. CI = Confidence interval; ICC = Intraclass correlation coefficient. τ = Random effects, ρ = correlation between random effects.

We present the results of the fitted model in Table S3. Critically, the results show the same three-way interaction between VMAC, Contingency Awareness, and Block detected in our experiments, with participants with higher contingency awareness showing a progressive increase in VMAC through the task. As noted earlier, the Contingency Awareness measure employed by Pearson et al. (2015) is a combination of two measures. Participants were first asked to report the color associated with high reward during the task, and then, they had to report their confidence level in their previous decision using a Likert scale. The confidence rating was then multiplied by +1 if participants correctly reported the contingency or by -1 if they failed to do so. The result of the previous analysis is difficult to interpret due to the combination of measures with different natures. A simpler interpretation may come from evidence that participants incorrectly reporting the contingency show no evidence of learning through the task. To test this possibility, we re-ran the above GLMM, but this time including as a predictor whether participants correctly reported the contingency or not. This predictor was coded as a dummy variable, assigning the value of 0 to participants who incorrectly answered the question and 1 to the rest of the participants. For instance, all predictors are interpreted for participants who reported to be unaware of the association.

**Table S4**

*Model summaries for re-analysis of Pearson et al. (2015) with contingency awareness.*

|  |  | | |
| --- | --- | --- | --- |
| *Predictors* | *Odds Ratios* | *CI* | *p* |
| (Intercept) | 0.06 | 0.04 – 0.11 | **<0.001** |
| VMAC | 1.06 | 0.86 – 1.31 | 0.592 |
| AC | 1.23 | 0.72 – 2.11 | 0.445 |
| Block | 5.58 | 2.45 – 12.70 | **<0.001** |
| Contingency | 1.22 | 0.69 – 2.14 | 0.497 |
| VMAC × Block | 0.91 | 0.74 – 1.12 | 0.383 |
| AC × Block | 0.99 | 0.59 – 1.64 | 0.963 |
| VMAC × Contingency | 0.94 | 0.75 – 1.18 | 0.609 |
| AC × Contingency | 1.28 | 0.72 – 2.29 | 0.405 |
| Block × Contingency | 0.72 | 0.29 – 1.75 | 0.464 |
| (VMAC × Block) × Contingency | 1.50 | 1.20 – 1.88 | **<0.001** |
| (AC × Block) × Contingency | 0.92 | 0.54 – 1.57 | 0.750 |
| **Random Effects** | | | |
| σ^2^ | 3.29 | | |
| τ_00_ | 0.55 _Intercept_ | | |
| τ_11_ | 0.03 _Block_ | | |
| τ_11_ | 0.55 _VMAC_ | | |
| τ_11_ | 0.90 _AC_ | | |
| ρ_01_ | 0.08 | | |
|  | -0.07 | | |
|  | 0.07 | | |
| ICC | 0.20 | | |
| N | 59 | | |
| Observations | 3523 | | |
| Marginal R^2^ / Conditional R^2^ | 0.137 / 0.312 | | |

The results of this analysis are presented in Table S4. Critically, we observed the same three-way interaction between VMAC, Contingency Awareness, and Block, confirming that learning was only observed for participants who correctly reported the contingency. To facilitate the interpretation of this result, we present model predictions in Figure S11.


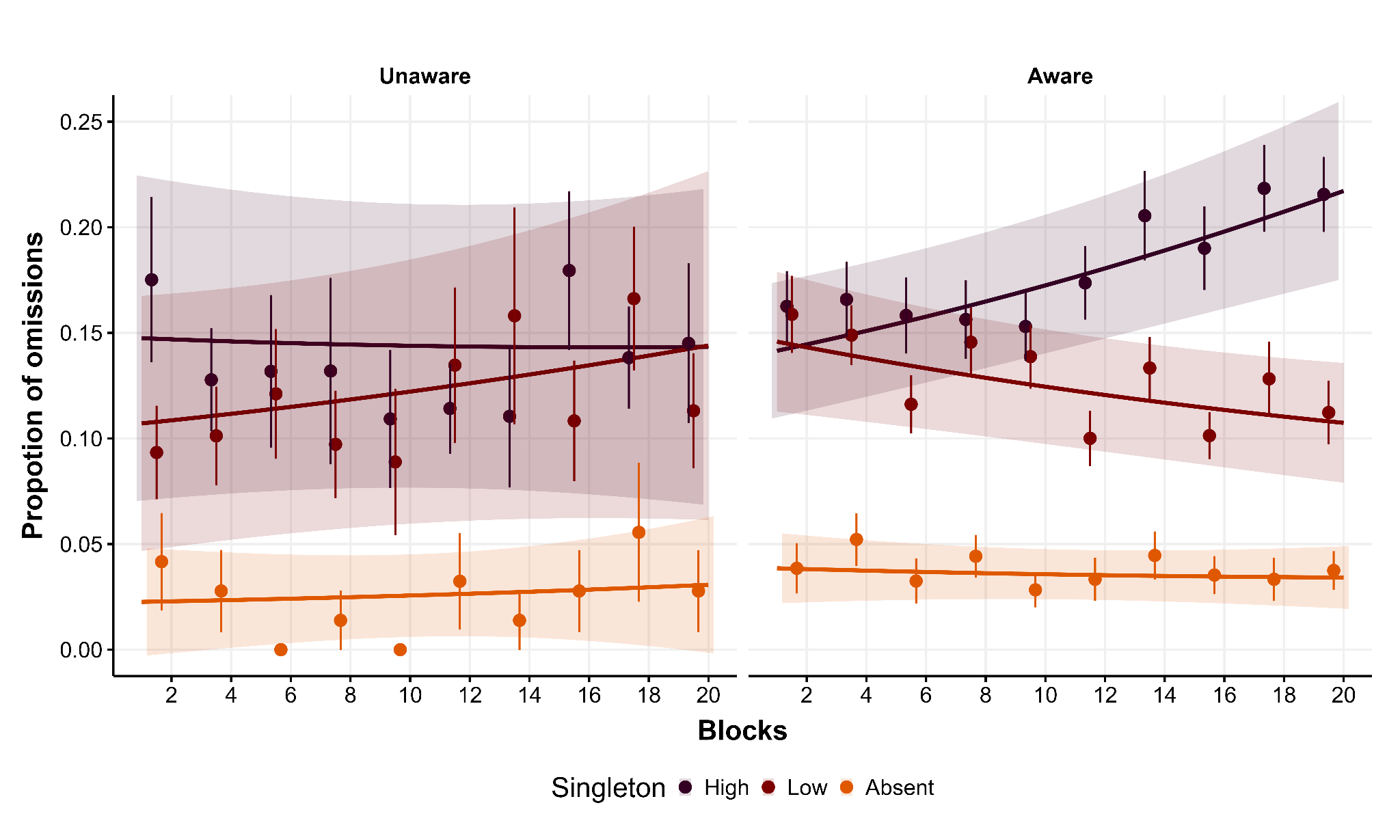


*Figure S11*. Model predictions as a function of awareness status and singletons across blocks. Lines represent the predicted conditional mean in the response scale, while shaded areas indicate the 95% CI. “Unaware” represents participants unable to report the correct color-reward contingency at the end of the task, while “Aware” refers to participants that correctly reported the contingency.

## Literature search strategy and effect size computation of the “Meta-analysis” section

*Search Strategy*

As we were interested in evaluating to what extent instructions modulate the size of VMAC in the one-stage paradigm developed by Le Pelley et al. (2015), we searched for all studies citing Le Pelley et al. (2015) in both the Web of Science and SCOPUS (date February 13^th^, 2025), resulting in a total of 221 studies. From those, we selected studies using the same one-stage paradigm. We employed the following criteria to define the one-stage paradigm:

- An arbitrary feature of a distracting stimulus is paired with a significant outcome.
- The associated feature is always task-irrelevant.
- The effect is measured using the Additional Singleton task (Theeuwes, 1992, 1994), where the manipulation is applied to task-irrelevant singleton distractors.
- The effect is measured “online” (while associations are being learned), and rewards are available.
- No other source information predicts the same outcome^^[[2]](#footnote-1)^^.

Based on those criteria, we select 39 studies (including our the two above reported experiments), in which 4 were excluded because did not report any statistics to compute an effect size. One further study was also excluded because participants were selected from a previous study that employed the same task as training (thus, we could not code the instructions nor the training length moderator).

*Computation of effect sizes*

We were interested in the comparison of the VMAC effect, which is defined as the difference between RTs or the proportion of omission trials with high- vs. low-value distractors (Rusz et al., 2020). As this contrast is often reported as a repeated measures ANOVA with a factor coding for the high-value or low-value distractor, or as a paired samples *t*-test, we used the *F* or the *t* statistic to calculate Cohen's *d_z_* (Lakens, 2013). We calculated the *d_z_* directly from the *t* statistic, and when we only had access to the *F* statistic, we transformed it to a *t* statistic following the rule *F* = *t*^2^ for factors with two levels. Then, we calculated Hedge’s *g_z_* (Hedges, 1981) employing the following formula:

$g_{z}= d_{z} \times(1 -\frac{3}{4N - 5})$

Sampling variances for each study (*V*_i_) were estimated as:

$V_{i}=\frac{1}{N_{i}}+\frac{g_{i}}{2N}$

where *N*_i_ and *g*_i_ represent the sample size and Hedges’s *g_z_* score of

each study, respectively (Cumming, 2012).

*Moderator analysis*

As explained in the main text, we coded three relevant moderators:

- The type of measure: RTs or Eye-tracking
- The type of instructions: Participants are instructed or not about the stimulus-outcome contingency
- Training length: Number of trials in training.

For the type of measure, there is a clear distinction between RTs and eye-tracking-based experiments. A previous meta-analysis detected that both types of measures could portray different sensibilities for measuring VMAC (Rusz et al., 2020), so we decided to include this moderator in our model. RT experiments require a manual response to stimuli within the search target (usually, the orientation of a line), and the manipulation of value is tied to 1) singleton distractor relevant feature and 2) performance (i.e., fast and accurate responses). In eye-tracking experiments, in contrast, participants are asked to attend to the target overtly and avoid looking at any of the distractors. When participants fixate on a distractor, the reward is omitted, and then they receive no outcome. Similar to RTs-based experiments, the outcome is a function of 1) the identity of the singleton distractor and 2) whether participants can look at the target while avoiding looking at the distractor. In those experiments, the main measure is the proportion of “omission trials”, defined as the proportion of trials in which the participant looks at the distractor before the target. Although there are other measures, such as saccade latencies (analogous to RTs-based measures), if more than one measure was available in an eye-tracking-based experiment, we selected the proportion of omission trials because it is the most widely used measure of VMAC.

Regarding instructions, we searched in the procedure section of all selected studies, looking for the explicit instructions given to participants before the experiment. If participants were informed about the contingency before the experiment began, we coded the experiment as “Instructions”; otherwise, we coded the study as “no instructions”. Lastly, regarding the length of training, we coded the overall number of trials employed in learning as a discrete-continuous number.

The hypothesis matrix for the moderators “Type of measure” and “Type of instructions” was set using deviation coding so that the intercept of our meta-regression model could still be interpreted as the overall VMAC effect across studies (Schad et al., 2020). In the case of “Training length”, we applied two transformations to enhance comparability with the present study. First, the moderator was centered so that the overall VMAC effect could be interpreted, taking as reference the training length of the present experiment (288 trials). Second, as in our analysis of VMAC, we log-transformed “Training length” so that the moderator codes for increments in the VMAC effect for each log increment in Training length. As in the LMM models employed in the main text, it is rarely the case that a temporal effect is completely linear. This apparent non-linearity is more explicit in Figure 5 of the main text, where we plot the predictions of our meta-regression model back-transforming Training length to the response scale. Note that using linear instead of log-transformed Training length does not affect the conclusion drawn from the present meta-analysis.

## ANOVA tables form results of Experiments 1 and 2

Here, we analyze the same results for Experiments 1 and 2 using ANOVAs for (G)LMMs models. This analysis performs the omnibus test for all predictors described in the main text; specific analysis as a function of relevant contrasts (VMAC and AC effects), which are critical for the interpretation of these tests, are presented in the main text.

**Table S5**

*ANOVA tables for (G)LMM results in Experiment 1 on RTs and Accuracy.*

|  | **RTs** | | | **Accuracy** | | |
| --- | --- | --- | --- | --- | --- | --- |
|  | $\chi^{2}$ | ***df*** | ***p*** | $\chi^{2}$ | ***df*** | ***p*** |
| **Distractor** | 50.726 | 2 | **<0.001** | 1.177 | 2 | 0.555 |
| **Block** | 129.024 | 1 | **<0.001** | 7.921 | 1 | **0.004** |
| **Distractor x Block** | 3.131 | 2 | 0.209 | 0.036 | 2 | 0.982 |

*Note.* Bold entries signal statistical significance.

**Table S6**

*ANOVA tables for (G)LMM results in Experiment 2 on RTs and Accuracy.*

|  | **RTs** | | | **Accuracy** | | |
| --- | --- | --- | --- | --- | --- | --- |
|  | $\chi^{2}$ | ***df*** | ***p*** | $\chi^{2}$ | ***df*** | ***p*** |
| **Distractor** | 197.118 | 2 | **<0.001** | 2.013 | 2 | 0.365 |
| **Block** | 358.081 | 1 | **<0.001** | 2.597 | 1 | 0.107 |
| **Group** | 0.0801 | 1 | 0.777 | 0.571 | 1 | 0.500 |
| **Distractor x Group** | 6.817 | 2 | **0.033** | 6.383 | 2 | **0.041** |
| **Distractor x Block** | 5.527 | 2 | 0.063 | 1.260 | 2 | 0.532 |
| **Block x Group** | 0.008 | 1 | 0.930 | 5.896 | 1 | **0.015** |
| **Distractor x Block x Group** | 3.032 | 2 | 0.220 | 2.3290 | 1 | 0.312 |

*Note.* Bold entries signal statistical significance.

## References

Cumming, G. (2012). *Understanding the new statistics: Effect sizes, confidence intervals, and meta-analysis*. Routledge/Taylor & Francis Group. <https://doi.org/10.4324/9780203807002>

Failing, M., & Theeuwes, J. (2017). Don’t let it distract you: How information about the availability of reward affects attentional selection. *Attention, Perception, & Psychophysics*, *79*(8), 2275-2298. <https://doi.org/10.3758/s13414-017-1376-8>

Garre-Frutos, F., Vadillo, M. A., González, F., & Lupiáñez, J. (2024). On the reliability of value-modulated attentional capture: An online replication and multiverse analysis. *Behavior Research Methods*, 1-18. <https://doi.org/10.3758/s13428-023-02329-5>

Hedges, L. V. (1981). Distribution theory for Glass's estimator of effect size and related estimators. *Journal of Educational Statistics, 6*(2), 107-128. <https://doi.org/10.3102/10769986006002107>

Lakens, D. (2013). Calculating and reporting effect sizes to facilitate cumulative science: a practical primer for t-tests and ANOVAs. *Frontiers in psychology, 4*, 863. <https://doi.org/10.3389/fpsyg.2013.00863>

Lakens, D. (2022). Sample size justification. *Collabra: psychology, 8*(1), 33267. <https://doi.org/10.1525/collabra.33267>

Mahlberg, J., Pearson, D., Le Pelley, M. E., & Watson, P. (2024). Prospective Distractor Information Reduces Reward-Related Attentional Capture. *Journal of Cognition, 7*(1), 50. <https://doi.org/10.5334/joc.375>

Meyen, S., Vadillo, M. A., von Luxburg, U., & Franz, V. H. (2023). No evidence for contextual cueing beyond explicit recognition. *Psychonomic Bulletin & Review*, 1-24. <https://doi.org/10.3758/s13423-023-02358-3>

Nunnally, J. C. (1978). *Psychometric theory* (2d ed). McGraw-Hill.

Parsons, S. (2022). Exploring reliability heterogeneity with multiverse analyses: Data processing decisions unpredictably influence measurement reliability. *Meta-Psychology*, *6*. <https://doi.org/10.15626/MP.2020.2577>

Pearson, D., Donkin, C., Tran, S. C., Most, S. B., & Le Pelley, M. E. (2015). Cognitive control and counterproductive oculomotor capture by reward-related stimuli. *Visual Cognition, 23*(1-2), 41-66. <https://doi.org/10.1080/13506285.2014.994252>

Parsons, S. (2021). Splithalf: Robust estimates of split half *reliability. Journal of Open Source Software, 6*(60), 3041.<http://dx.doi.org/10.21105/joss.03041>

Rusz, D., Le Pelley, M. E., Kompier, M. A. J., Mait, L., & Bijleveld, E. (2020). Reward-driven distraction: A meta-analysis. *Psychological Bulletin*, *146*(10), 872-899. <https://doi.org/10.1037/bul0000296>

Schad, D. J., Vasishth, S., Hohenstein, S., & Kliegl, R. (2020). How to capitalize on a priori contrasts in linear (mixed) models: A tutorial. J*ournal of memory and language, 110,* 104038. <https://doi.org/10.1016/j.jml.2019.104038>

Shanks, D. R. (2017). Regressive research: The pitfalls of post hoc data selection in the study of unconscious mental processes. *Psychonomic Bulletin & Review*, *24*, 752-775. <https://doi.org/10.3758/s13423-016-1170-y>

Vadillo, M. A., Malejka, S., Lee, D. Y., Dienes, Z., & Shanks, D. R. (2022). Raising awareness about measurement error in research on unconscious mental processes. *Psychonomic Bulletin & Review*, *29*(1), 21-43. <https://doi.org/10.3758/s13423-021-01923-y>

1. In the context of the VMAC x Block interaction, the VMAC effect calculated in this power analysis is conditional to the higher-order interaction. In other words, as the Block predictor is centered, the VMAC effect here refers to the VMAC effect in block six/seven, which may be a more conservative power calculation given that VMAC increases over time. Taking the averaged VMAC effect across blocks would result in an increase in statistical power. [↑](#footnote-ref-0)
2. Some studies have shown that prospective information about an upcoming outcome significantly hampers the VMAC effect (Failing and Theeuwes, 2017; Malghber et al., 2024). Consequently, we only selected contrasts where features of singleton distractors are the only source of information about the outcome. When this is manipulated between different stages, we selected the statistics of the stage in which only the color singleton is predictive (Failing & Theewues, 2017; Malghber et al., 2024).- [↑](#footnote-ref-1)
